# Concerted regulation of npc2 binding to endosomal/lysosomal membranes by bis(monoacylglycero)phosphate and sphingomyelin

**DOI:** 10.1371/journal.pcbi.1005831

**Published:** 2017-10-30

**Authors:** Giray Enkavi, Heikki Mikkolainen, Burçin Güngör, Elina Ikonen, Ilpo Vattulainen

**Affiliations:** 1 Laboratory of Physics, Tampere University of Technology, Tampere, Finland; 2 Department of Physics, University of Helsinki, Helsinki, Finland; 3 Department of Anatomy, Faculty of Medicine, University of Helsinki, Helsinki, Finland; 4 Minerva Foundation Institute for Medical Research, Helsinki, Finland; 5 Memphys—Center for Biomembrane Physics, Odense, Denmark; University of Maryland School of Pharmacy, UNITED STATES

## Abstract

Niemann-Pick Protein C2 (npc2) is a small soluble protein critical for cholesterol transport within and from the lysosome and the late endosome. Intriguingly, npc2-mediated cholesterol transport has been shown to be modulated by lipids, yet the molecular mechanism of npc2-membrane interactions has remained elusive. Here, based on an extensive set of atomistic simulations and free energy calculations, we clarify the mechanism and energetics of npc2-membrane binding and characterize the roles of physiologically relevant key lipids associated with the binding process. Our results capture in atomistic detail two competitively favorable membrane binding orientations of npc2 with a low interconversion barrier. The first binding mode (*Prone*) places the cholesterol binding pocket in direct contact with the membrane and is characterized by membrane insertion of a loop (V59-M60-G61-I62-P63-V64-P65). This mode is associated with cholesterol uptake and release. On the other hand, the second mode (*Supine*) places the cholesterol binding pocket away from the membrane surface, but has overall higher membrane binding affinity. We determined that bis(monoacylglycero)phosphate (bmp) is specifically required for strong membrane binding in *Prone mode*, and that it cannot be substituted by other anionic lipids. Meanwhile, sphingomyelin counteracts bmp by hindering *Prone mode* without affecting *Supine mode*. Our results provide concrete evidence that lipids modulate npc2-mediated cholesterol transport either by favoring or disfavoring *Prone mode* and that they impose this by modulating the accessibility of bmp for interacting with npc2. Overall, we provide a mechanism by which npc2-mediated cholesterol transport is controlled by the membrane composition and how npc2-lipid interactions can regulate the transport rate.

## Author summary

Cholesterol plays essential structural and functional roles in all vertebrate cells. Abnormalities in cholesterol metabolism are associated with severe and wide-spread diseases. Cholesterol efflux is one of the essential steps in its metabolism and is mediated by Niemann-Pick Protein C2 (npc2) in endosomes and lysosomes. Mutations in npc2 result in the fatal genetic disease, called Niemann-Pick C disease, characterized by neuronal degeneration resulting in early death. We performed an extensive set of atomistic molecular dynamics simulations employing enhanced sampling approaches to investigate how npc2 mediates cholesterol transport and the roles of membrane lipids in the process. We show that the binding of npc2 to endosomal/lysosomal membranes and the consequent trafficking of cholesterol are concertedly regulated by bis(monoacylglycero)phosphate and sphingomyelin. Moreover, our results explain the molecular mechanism of experimentally observed affect of lipids on cholesterol transport rate. In essence, our findings provide key insights into the regulation of the lysosomal and endosomal cholesterol trafficking by npc2 and reveal key lipid-protein interactions.

## Introduction

Cholesterol, ubiquitously present in all vertebrate cells, regulates the structure and permeability of cellular membranes [1, 2]. It comprises typically 20–25 mol % of all lipids in the plasma membrane [1] and acts as a precursor for many bioactive molecules such as steroid hormones, bile acids, oxysterols, and vitamin D [2]. While cholesterol is vital for health, its excessive accumulation results in pathologies, such as cardiovascular diseases, the complications of which account for about 30% of deaths globally [2]. Because most cells cannot catabolize cholesterol, its efflux is crucial to prevent cholesterol overloading [2].

There is a network of cellular signaling and transport systems that tightly controls cholesterol trafficking [1]. One of the key proteins involved in cholesterol efflux from late endosomes/lysosomes is Niemann-Pick Protein C2 (npc2), an intralysosomal and secretory protein found in epididymal fluid, milk, plasma, and the bile [3]. Mutations in npc2 or its transmembrane partner npc1 result in accumulation of lipids such as unesterified cholesterol and sphingolipids [4, 5]. This fatal genetic lysosomal storage condition called Niemann-Pick C disease results in progressive neuronal degeneration in the brain and early death [6, 7].

In the late endosome/lysosome, a “tag team duo” composed of npc1 and npc2 is responsible for egress of endocytosed cholesterol [4, 8]. npc2 captures cholesterol from the internal membranes of late endosomes/lysosomes and transfers it to npc1 [9, 10] for cholesterol egress from these compartments [5]. There has been recent resurgence of npc1 structures characterized by crystallography and cryo-electron microscopy [11–15], reviving interest in molecular investigation of late endosomal/lysosomal cholesterol trafficking. These structural studies have been complemented by computational investigations focused on the cholesterol transfer process between the N-terminal domain of npc1 and npc2 [16, 17], as well as sterol-binding to npc2 [18].

Meanwhile, npc2 also works independently of npc1 [19]. In particular, it has been shown that normalizing abca1 expression can bypass the effects of npc1 mutations, but not those of npc2 mutations [20], highlighting the importance of the npc1-independent cholesterol transport by npc2. In this work, we investigate the npc1-independent mechanism, namely the direct specific binding of npc2 onto the internal late endosomal/lysosomal membranes to cargo cholesterol between them [19, 21].

npc2 mainly functions in the acidic environment of late endosomes and lysosomes. These organelles manifest a multivesicular appearance due to the presence of a unique anionic phospholipid called bis(monoacylglycero)phosphate (bmp), also known as lysobisphosphatidic acid (lbpa) [22]. bmp is abundant within the internal membranes of late endosomes/lysosomes, yet it is absent from the cytoplasmic leaflet of the limiting membrane [22]. The intraluminal vesicles are the sites of sphingolipid degradation, with sphingomyelin (sm) representing the most abundant sphingolipid [23]. When bound to a membrane, npc2 interacts specifically with bmp, which modulates its efficiency in cholesterol transport [21, 24–26]. On the other hand, sm strongly inhibits cholesterol transfer by npc2 [27]. Nevertheless, the molecular interactions between npc2 and different lipid components, and in particular how bmp and sm contributes to this dual regulation of npc2-dependent cholesterol transport remain unclear.

We used atomistic molecular dynamics (MD) simulations to perform an extensive investigation of npc2-membrane interactions in a variety of different membrane lipid mixtures of neutral (1-palmitoyl-2-oleoyl-*sn*-glycero-3-phosphocholine (popc), N-palmitoyl-D-*erythro*-sphingosylphosphorylcholine (sm)) and charged (s-s 2,2’-dioleoyl lysobisphosphatidic acid (bmp), 1,2-dioleoyl-*sn*-glycero-3-phosphatidylglycerol (dopg)) lipids together with cholesterol (chol). We determined the lipid-dependent membrane-binding free energies using *well-tempered metadynamics* (wt-mtd) on three *collective variables* (cvs) that define the orientation and the position of npc2 with respect to the membrane. The results highlight that i) npc2 binds charged membranes favorably; ii) npc2 binds membranes in two different competitively favorable orientations; iii) bmp is decisive for strong npc2 binding in the orientation that places the cholesterol-binding pocket of npc2 in direct contact with the membrane; and iv) sm hinders the formation of the aforementioned orientation. We characterized specific lipid-protein interactions in different orientations to understand the bases of the observed binding and inhibition mechanisms induced by bmp and sm. Altogether, the results provide an atom-scale picture for npc2-membrane binding and insights into how the membrane composition modulates the efficiency of npc2-dependent cholesterol transport.

## Materials and methods

We considered a total of 8 different membrane systems (see Table 1) composed of bmp, chol, sm, dopg, and popc. For 5 of these membrane systems (systems 1, 3–5, 7 in Table 1), we performed unbiased simulations with npc2 in its cholesterol-bound (npc2^chol-bound^) and *apo* (npc2^*apo*^) forms. Seven 400 ns long repeats were performed, each starting from a randomly chosen independent initial configuration, adding up to a total of 70 unbiased simulations. We also performed (biased) free energy calculations using well-tempered metadynamics (wt-mtd) [28] for all membrane systems except System 4 with npc2^chol-bound^ and npc2^*apo*^. The total simulation time for unbiased and biased simulations add up to 28 *μ*s and ∼148 *μ*s, respectively.

**Table 1:** Description of the simulated systems and a summary of the main results as to binding processes.

System	Membrane Composition/(mol%)					No. observations				Free Energy/(kJ/mol)					
						chol-bound		*apo*		chol-bound			*apo*		
	bmp	chol	sm	dopg	popc	*Prone*	*Supine*	*Prone*	*Supine*	*Prone*	*Supine*	Membrane Associated	*Prone*	*Supine*	Membrane Associated
1	0	10	0	0	90	0	0	0	0	-0.5±0.7	1.1±0.6	0.9±0.5	0.2±0.5	-0.8±0.5	0.7±0.4
2	0	35	0	0	65	*not performed*				1.9±0.5	1.4±0.5	1.4±0.4	0.6±0.4	-0.4±0.3	0.7±0.3
3	0	10	20	0	70	0	1	0	0	-0.4±0.5	-0.2±0.5	-0.1±0.4	0.4±0.5	-0.5±0.5	0.6±0.4
4	100	0	0	0	0	1	6	1	6	*not performed*					
5	20	10	0	0	70	1	6	0	0	-11.5±0.8	-11.9±0.7	-10.6±0.6	-10.5±0.8	-12.0±0.8	-10.1±0.7
6	20	35	0	0	45	*not performed*				-10.8±0.8	-14.9±0.7	-12.3±0.6	-10.0±0.6	-12.4±0.6	-10.1±0.6
7	20	10	20	0	50	0	5	0	4	-7.5±1.0	-12.2±1.0	-9.7±0.9	-8.8±0.8	-11.9±0.7	-10.0±0.8
8	0	10	0	20	70	*not performed*				-6.2±0.7	-8.5±0.7	-6.6±0.5	-7.6±0.7	-9.9±0.7	-8.1±0.7

Membrane content is given in terms of molar fractions for a mixture of bmp:chol:sm:dopg:popc. No. observations lists the number of unbiased simulations out of 7 repeats, in which *Prone* or *Supine mode* formed. Free energy contribution to binding for each orientation is also shown based on the free energy calculations (see S1 Text for the details on how the values are obtained). The phrase *not performed* specifies that the simulations were not performed for that particular membrane composition.

The preparation of all systems including the membrane mixtures is described in detail in S1 Text. All simulations were performed using gromacs 5.0 [29] employing the Amber ff99sb-ildn force field [30] for the protein, the Slipids force field [31] for the lipids, and the tip3p model for water [32].

The equations of motion were integrated using a leap-frog algorithm with a 2 fs time step. All bonds were constrained using the lincs algorithm [33]. Long-range electrostatic interactions were treated by the smooth particle mesh Ewald scheme (spme) [34, 35] with a real-space cutoff of 1.0 nm, a Fourier spacing of 0.16 nm, and a fourth-order interpolation. The van der Waals interactions were treated with a Lennard-Jones potential with a cutoff of 1.0 nm. Long-range dispersion corrections were applied for energy and pressure [36].

Before production runs, successive steepest descent minimizations were carried out, followed by short equilibration simulations in the *NVT* and *NpT* ensembles at a temperature of 310°K using the v-rescale thermostat [37] with a time constant of 0.1 ps. The pressure was kept at 1 atmosphere using the Berendsen barostat [38] with a time constant of 0.5 ps.

All production simulations were performed in the *NpT* ensemble. Protein, solvent (water and ions), and lipids were coupled to separate temperature baths at 310°K using the Nosé-Hoover thermostat [39, 40] with a time constant of 0.5 ps. Pressure was kept at 1 atmosphere with a time constant of 10 ps and a compressibility of 4.5 × 10^−5^ bar^−1^ using the Parrinello-Rahman barostat [41, 42] semi-isotropically for membrane-containing systems and isotropically for others.

For the free energy calculations, all biased simulations employed gromacs 5.0.4 [29] patched with plumed 2.1 [43]. Details of these free energy computations are discussed in S1 Text, which also includes a more detailed description of the simulation protocol.

## Results

### npc2 binds anionic bmp-containing membranes in two distinct binding modes

**Fig 1:**
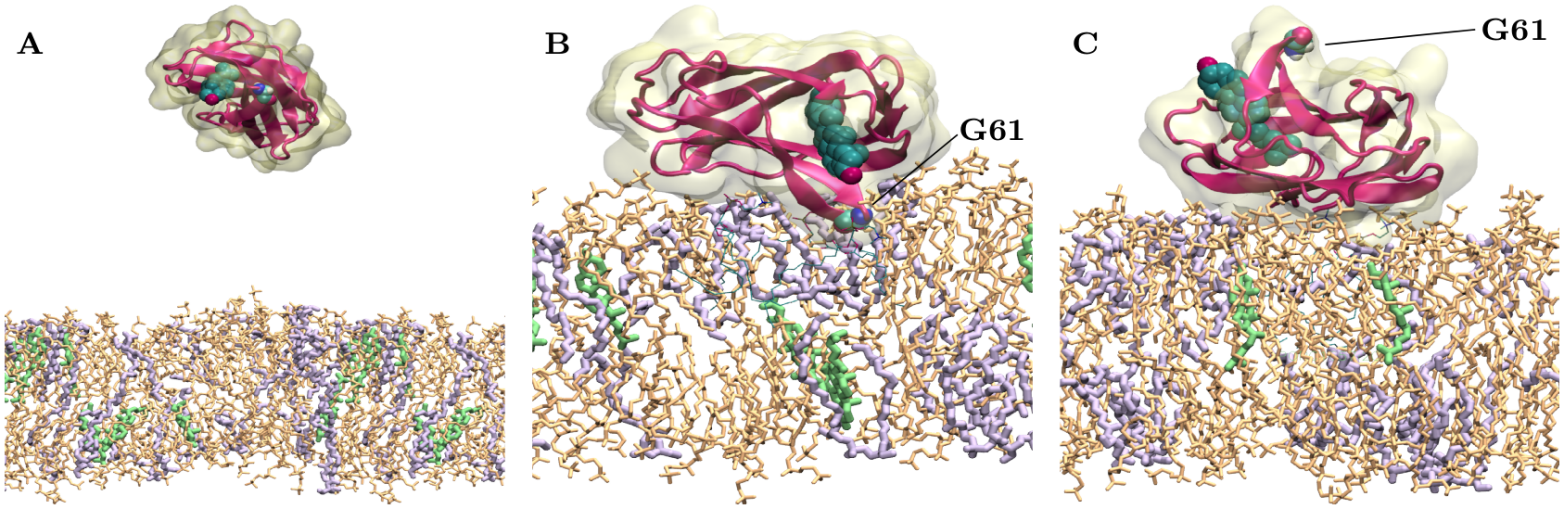
Snapshots of simulated system with a lipid composition of bmp:chol:sm:dopg:popc = 20:10:0:0:70 (System 5). A) A typical starting configuration for npc2 in the water phase, above the membrane. B) Final configuration after a simulation of 400 ns in a case, where the cholesterol-binding pocket faces the membrane (*Prone mode*). C) Final configuration of a simulation, where the cholesterol-binding pocket faces the water phase (*Supine mode*). The residue G61 residing at the opening of the binding pocket is highlighted. Color code for lipids: bmp (violet), chol (green), popc (light brown). For clarity’s sake, water is not shown.

To investigate the role of bmp, sm, and chol in npc2–membrane binding, we prepared a number of membrane models with different lipid compositions listed in Table 1. For most of the membrane systems, we first carried out unbiased md simulations, where we initially placed *apo* (npc2^*apo*^) [44] or chol-bound npc2 (npc2^chol-bound^) [3] above the membrane surface in seven different, randomly chosen orientations such that the minimum *z*-distance between the protein and the membrane surface is 30–35Å (see Fig 1A for a typical initial configuration). In these unbiased simulations, the membranes containing bmp (bmp:chol:sm:dopg:popc = 100:0:0:0:0 (System 4), 20:10:0:0:70 (System 5), and 20:10:20:0:50 (System 7)) resulted in spontaneous association of npc2 with the membrane in less than 60 ns (Fig 1B and 1C). For comparison, if the membrane considered did not include bmp (bmp:chol:sm:dopg:popc = 0:10:0:0:90 (1) and 0:10:20:0:70 (System 3)), then no npc2 binding was observed. The only exception was chol:sm:popc = 10:20:70, where one out of 14 simulations resulted in npc2 binding to the membrane.

Monitoring the distance between the membrane surface and the residue at the opening of the cholesterol-binding pocket (G61), we observed that npc2 binds the membranes in two distinct modes based on the membrane-adsorbed state: membrane binding orientation 1 (*Prone mode*, Fig 1B), in which the cholesterol-binding pocket is in direct contact with the membrane surface and G61 is inserted into the membrane; and orientation 2 (*Supine mode*, Fig 1C), in which G61 is facing away from the membrane surface. *Supine mode*, formed by rotating the protein ∼180° along its long axis with respect to *Prone mode*, places the cholesterol-binding pocket away from the membrane to face the water phase. In these simulations, *Supine mode* is observed more frequently than *Prone mode* for both npc2^*apo*^ and npc2^chol-bound^ (Table 1) and the orientations do not interconvert within 400 ns. Table 1 shows the number of times each orientation is observed for each membrane system based on the location of G61 with respect to the membrane surface.

Based on the results, one can conclude that i) the simulations reveal two functional binding modes with favorable energetics, and ii) the npc2-membrane interaction is so strong that once the binding has taken place, the protein is trapped in one of the two local minima and the orientation does not change during the simulation time scale of 400 ns.

### npc2 changes binding orientation by rocking around its long axis

Characterizing the aforementioned membrane binding modes both energetically (to compute the free energy of binding) and structurally (to determine the mechanism of binding) requires extensive sampling of the relevant degrees of freedom. To this end, we used a combination of well-tempered metadynamics (wt-mtd) [28], umbrella sampling, and bias exchange [45]. A similar combination of wt-mtd and umbrella sampling was previously used in coarse-grained simulations [46].

**Fig 2:**
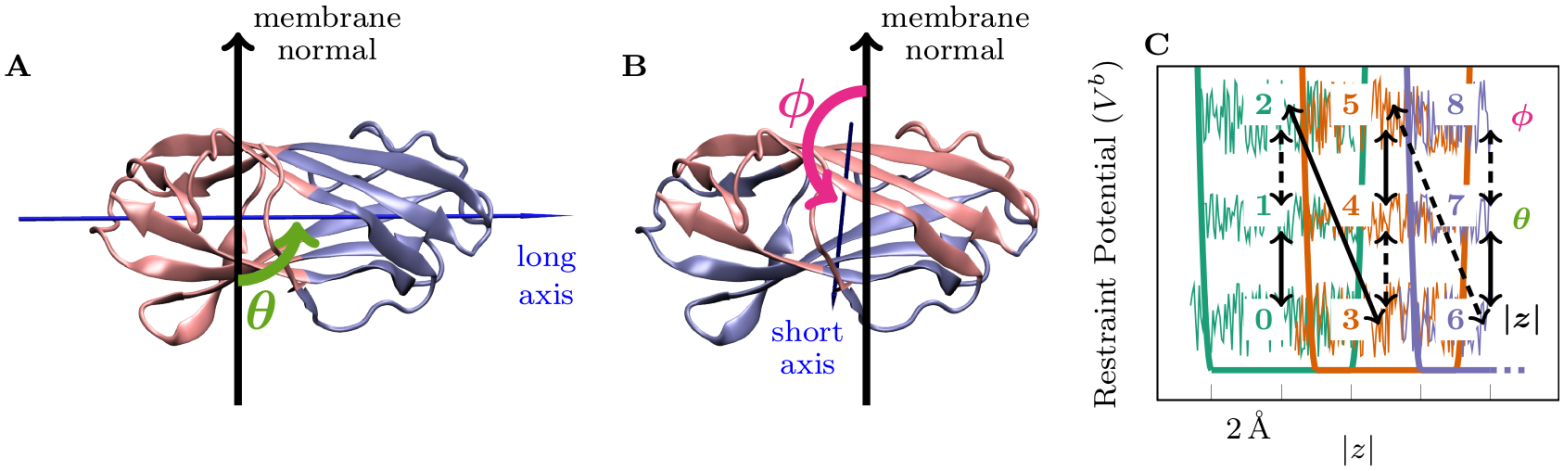
The collective variables and the sampling approach. npc2 structure overlaid with vectors showing the long (A) and the short (B) axes of the protein and the collective variables *θ* (A) and *ϕ* (B). The membrane normal defined as the vector between the com of P atoms of the upper and the lower leaflets of the membrane is represented with a solid arrow. A) The long axis is defined as a vector connecting the C_*α*_ com of residues 1–6, 19–38, 55–69, 93–109, 123–130 (pink), and that of residues 7–18, 39–54, 70–92, 110–122 (blue). B) The short axis is defined as a vector connecting the C_*α*_ com of residues 1–6, 13–45, 75–94, 124–130 (pink), and and that of residues 7–12, 46–74, 95–123 (blue). C) A schematic representation of the sampling approach. The numbers indicate the replica indices (*i*). Three separate wt-mtd simulations biasing |*z*| (*i* = 0, 3, …), *θ* (*i* = 1, 4, …), and *ϕ* (*i* = 2, 5, …) were performed within each window (*w*, shown in different colors) flanked by half harmonic restraints. Bias exchanges were attempted between simulations *i* and *i* + 1 in even (solid arrows) and odd pairings (dashed arrows). For a detailed discussion of the sampling procedure, see S1 Text.

A schematic representation of our sampling approach is shown in Fig 2 and the details can be found in S1 Text. Briefly, we defined three collective variables (cvs) that describe the position and the orientation of the protein with respect to the membrane:

1. |*z*|: projection of the distance between the center of mass (com) of the P atoms of the membrane upper leaflet and the com of C_*α*_ atoms of the protein on to the *z*-axis, the coordinate along the membrane normal direction. This collective variable describes the npc2-membrane distance.
2. *θ*: angle between the long axis of the protein and the membrane normal (Fig 2A), thereby describing the principal orientation of npc2 with respect to the membrane surface.
3. *ϕ*: the angle between the short axis of the protein and the membrane normal (Fig 2B). Together, the angles *θ* and *ϕ* uniquely define the protein orientation at the membrane surface.

We divided the |*z*| range that extends from the membrane surface to bulk solvent into 4Å windows that overlap 1Å with each other using half harmonic restraints. Within each window, we performed three separate wt-mtd simulations [28], in each biasing one of |*z*|, *θ*, or *ϕ*. This adds up to 51–54 200 ns-long simulations (17–18 windows) for each system. To further improve the sampling efficiency, we coupled the neighboring simulations through the bias exchange scheme [45] as shown in Fig 2. wt-mtd helps overcome barriers along |*z*|, *ϕ*, and *θ* by flattening the underlying free energy landscape, while half-harmonic restraints keep sampling within manageable blocks. Besides, bias exchange improves the statistical efficiency and the convergence of the free energy estimations. This approach allows reconstruction of potential of mean force (pmf) profiles for complex systems using a reweighting scheme that combines a time-independent locally-converging free energy estimator for metadynamics [47] and a non-parametric variant of the weighted histogram analysis method [48], discussed in S1 Text.

**Fig 3:**
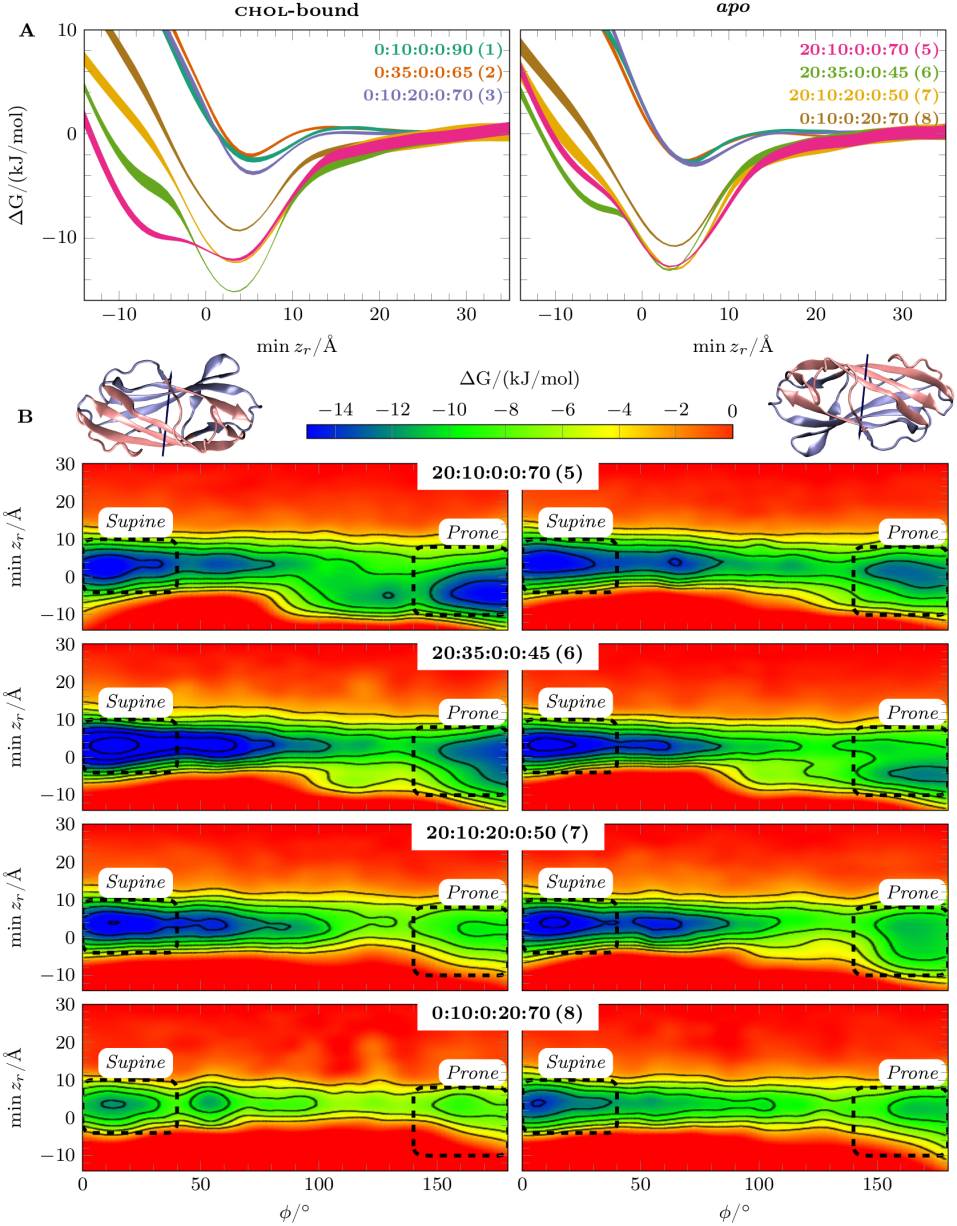
The free energy surfaces for npc2 binding to membranes. A) Potential of mean force (pmf) profiles shown as a function of min *z*_*r*_, which stands for the minimum of the protein C_*α*_ *z*-coordinates for the residue *r*. The origin (*z* = 0) is set to com_*z*_ of the upper leaflet P atoms. B) pmf surfaces shown as a function of min *z*_*r*_ and *ϕ* for anionic membranes. Contours were plotted every 2 kJ/mol increments. The two binding orientations are marked with dashed lines. The thumbnail images of npc2 structure for the corresponding orientations are shown on the upper left corner for *Prone mode* and on the upper right corner for *Supine mode*. See S9 Fig for the local errors and S1 Fig for the pmf projected onto |*z*|. The labels indicate membrane content in molar fractions for a mixture of bmp:chol:sm:dopg:popc, and the corresponding system numbers (Table 1) are provided in parentheses. The local errors are given in S9 Fig.

Based on these free energy simulations, we reconstructed one-dimensional (1d) and two-dimensional (2d) pmf profiles for each simulated system as functions of the biased collective variables (see S1 Text for details). All reconstructed free energy surfaces and their errors are given separately for the anionic and neutral membranes. 1d pmfs as a function of |*z*| are given in S1A Fig. 2d pmfs and their local errors for |*z*| vs *ϕ* are given in S1B and S2 Figs for anionic membranes and in S3B and S4 Figs for neutral membranes. The pmfs for |*z*| vs *θ* are given in S5B and S6 Figs for anionic membranes and in S7B and S8 Figs for neutral membranes. To capture the depth of insertion into the membrane, we set the origin (*z* = 0) to the *z*-coordinate of the center of mass (com_*z*_) of the upper leaflet P atoms and reconstructed the pmfs also as functions of the minimum of the protein C_*α*_ *z*-coordinates, which is here denoted as min *z*_*r*_, *r* being the residue number. See Fig 3A for 1d pmfs, Fig 3B and S9 Fig for 2d pmfs for charged membranes, and S10 and S11 Figs for 2d pmfs for neutral membranes.

Here, we discuss the general features of npc2-membrane binding. In agreement with the above-discussed unbiased simulations, the results show enhanced npc2 binding only to anionic membranes rich in bmp. In neutral membranes without bmp (bmp:chol:sm:dopg:popc = 0:10:0:0:90 (1), 0:35:0:0:65 (System 2), and 0:10:20:0:70 (System 3)), the affinity for the membranes is low (Fig 3A and S1A Fig). The binding to neutral membranes is unspecific in terms of *ϕ* (S10 and S3 Figs) and *θ* (S7 Fig), as no particular binding orientation can be identified. In anionic membranes (bmp:chol:sm:dopg:popc = 100:0:0:0:0 (System 4), 20:10:0:0:70 (System 5), 20:35:0:0:45 (System 6), 20:10:20:0:50 (System 7), and 0:10:0:20:70 (System 8)), on the other hand, we observe the two distinct high-affinity binding orientations (*ϕ* >∼140° and *ϕ* <∼40° in *Prone* and *Supine* modes, respectively) with overall enhanced affinity in *Supine mode* (Fig 3B and S1B Fig). The pmf profiles (S5 Fig) reveal a single specific high affinity binding orientation for the anionic membranes in terms of *θ* in a range of about 80° < *θ* < 100°.

*Supine mode* has generally lower free energies than *Prone mode* and is more plastic as indicated by the wider distribution of *ϕ* in *Supine mode* (Fig 3 and S1 Fig). This also suggests that *Supine mode* is more flexible in terms of membrane-interacting residues and may be better described as a membrane-adsorbed state rather than as a specific membrane-bound state. On the other hand, *Prone mode* is characterized by a tighter distribution of *ϕ* and deeper insertion into the membrane (Fig 3B). This deeply inserted state, also apparent in 1d pmf profiles (Fig 3A), is uniquely associated with *Prone mode* and forms by the membrane insertion of a loop (V59-M60-G61-I62-P63-V64-P65) at the opening of the cholesterol-binding pocket. From here on, we refer to this hydrophobic loop as the *membrane insertion loop* (mil).

The position of the cholesterol-binding pocket and three residues (F66, V96, and T100) that act as gates in sterol transfer [18] with respect to the membrane surface, as well as the membrane insertion of the loop strongly suggest that *Prone mode* is the conductive state for cholesterol uptake/release.

Because the interconversion between *Prone* and *Supine* modes can determine the efficiency of cholesterol uptake/release, it is potentially an important process for npc2 function. The interconversion can take place by protein detaching from the membrane and reattaching in either of the orientations. This process requires surpassing the barrier equal to the free energy of membrane binding, which in BMP-containing membranes is typically 7 − 15 kJ/mol and hence quite large (Table 1). Alternatively, the protein could roll around its long axis without detaching from the membrane. The latter process has a barrier of about 6 − 7 kJ/mol (about 2.5 to 3 *k*_B_*T*) along *ϕ* (Fig 3B), which is quite low compared to thermal energy.

We conclude that npc2 changes the binding orientation by rocking around its long axis while staying adsorbed on the membrane surface.

### bmp promotes formation of *Prone mode* and is required for membrane anchoring

bmp is known to improve npc2-mediated cholesterol transfer between membranes [27]. To investigate the effect of bmp on the free energy of npc2-membrane binding, we performed membrane binding free energy simulations for both npc2^*apo*^ and npc2^chol-bound^ in the presence of bmp. We focus our discussion here on the investigations with the anionic bmp:chol:sm:dopg:popc = 20:10:0:0:70 (System 5) mixture together with the neutral membranes that do not contain bmp (Table 1). Both npc2^*apo*^ and npc2^chol-bound^ bind strongly in two specific binding orientations to bmp membranes, the binding free energies being around or larger than Δ*G* = −11 kJ/mol. In neutral membranes, the binding affinity is much lower (Δ*G* = −3 to −4 kJ/mol) and manifests unspecific adsorption in terms of *ϕ* and *θ* as mentioned above (S10, S3 and S7 Figs). The 2d pmf in Fig 3B (1^st^ row) shows that *Prone mode* is almost as favorable as *Supine mode*, especially when npc2 is cholesterol-bound. Moreover, bmp also promotes membrane anchoring by the *membrane insertion loop* (mil) as evidenced by deeper inserted states captured in the pmf profiles.

In essence, bmp does not only improve npc2 adsorption onto the membrane surface, but it also promotes the binding mode (*Prone*) in which cholesterol uptake and release can take place, along with membrane anchoring by mil.

### bmp’s effect on npc2-membrane binding is not only due to its negative charge

The electrostatic potential profiles (S12 Fig) lack a substantial difference between membrane compositions that could explain how and why npc2, which has +4*e* at pH = 5 (see S1 Text), favors negatively charged bmp. To further test whether the effects of bmp on npc2-membrane binding are specific or can simply be attributed to its negative charge, we performed free energy simulations, where bmp is substituted with dopg (bmp:chol:sm:dopg:popc = 0:10:0:20:70 (System 8)). Phosphatidylglycerol (pg) lipids are not only negatively charged, but they are also precursors in bmp synthesis [49]. Although the negative charge of dopg clearly enhances membrane adsorption (Δ*G* = −9 to −10 kJ/mol) when compared to the neutral membranes, the dopg membrane has ∼ 3 kJ/mol lower affinity for both npc2^*apo*^ and npc2^chol-bound^ when compared to bmp-containing membranes (Fig 3A and S1A Fig). Moreover, dopg does not favor npc2-binding in *Prone mode* as strongly as bmp, and does not foster the penetration of the *membrane insertion loop* into the membrane (Fig 3B; last row).

Because pg cannot reproduce the effects of bmp, we conclude that specific bmp-npc2 interactions are important in both membrane binding and cholesterol uptake/release.

### npc2-membrane binding is cholesterol dependent

The higher cholesterol concentration is expected to shift the equilibrium between the npc2^*apo*^ and npc2^chol-bound^ towards the cholesterol-bound state. We further hypothesized that cholesterol concentration in the membrane can also affect npc2-membrane binding properties. Increasing the cholesterol concentration from 10 to 35 mol% in the presence of 20 mol% bmp (bmp:chol:sm:dopg:popc = 20:10:0:0:70 (System 5) and 20:35:0:0:45 (System 6); see Table 1) enhances membrane adsorption for npc2^chol-bound^ by ∼3 kJ/mol (at min *z*_*r*_ = ∼ 3Å) without affecting that of npc2^*apo*^. However, higher cholesterol concentration has the opposite effect for the deeper inserted binding mode of npc2 (*Prone mode*). The deeper inserted state (around min *z*_*r*_ = −6 Å) is destabilized by ∼5 kJ/mol for npc2^chol-bound^, while it is stabilized by ∼3 kJ/mol for npc2^*apo*^ (Fig 3A). The same effect can be more clearly seen in 2d pmf profiles (Fig 3B, 1^st^ and 2^nd^ rows). Comparing 10 mol% and 35 mol% chol membranes, the minimum for *Prone mode* shifts up towards the membrane surface and its free energy is increased in npc2^chol-bound^, while it is shifted downwards in npc2^*apo*^. In other words, while both npc2^*apo*^ and npc2^chol-bound^ adsorb on the bmp-rich membranes with high affinity, the higher chol concentration shifts the free energy minimum towards the deeper inserted *Prone mode* in npc2^*apo*^ and away from it in npc2^chol-bound^.

This result implies that the cholesterol levels in the internal membranes of late endosomes/lysosomes can affect the rate of npc2-mediated cholesterol transport by affecting the equilibrium between *Prone* and *Supine* modes, taking into account that deeper inserted *Prone mode* is conductive for cholesterol uptake and release.

### Sphingomyelin impairs npc2-membrane binding in *Prone mode*

sm is one of the components of the suborganelle fractions of late endosomes [22]. Furthermore, sm has been shown to strongly inhibit cholesterol-transport by npc2 [27]. To investigate if and how sm affects npc2-membrane binding, we performed unbiased and free energy simulations to membranes containing 20 mol% sm in the presence and absence of bmp (bmp:chol:sm:dopg:popc = 20:10:20:0:50 (System 7) and 0:10:20:0:70 (System 3); see Table 1).

**Fig 4:**
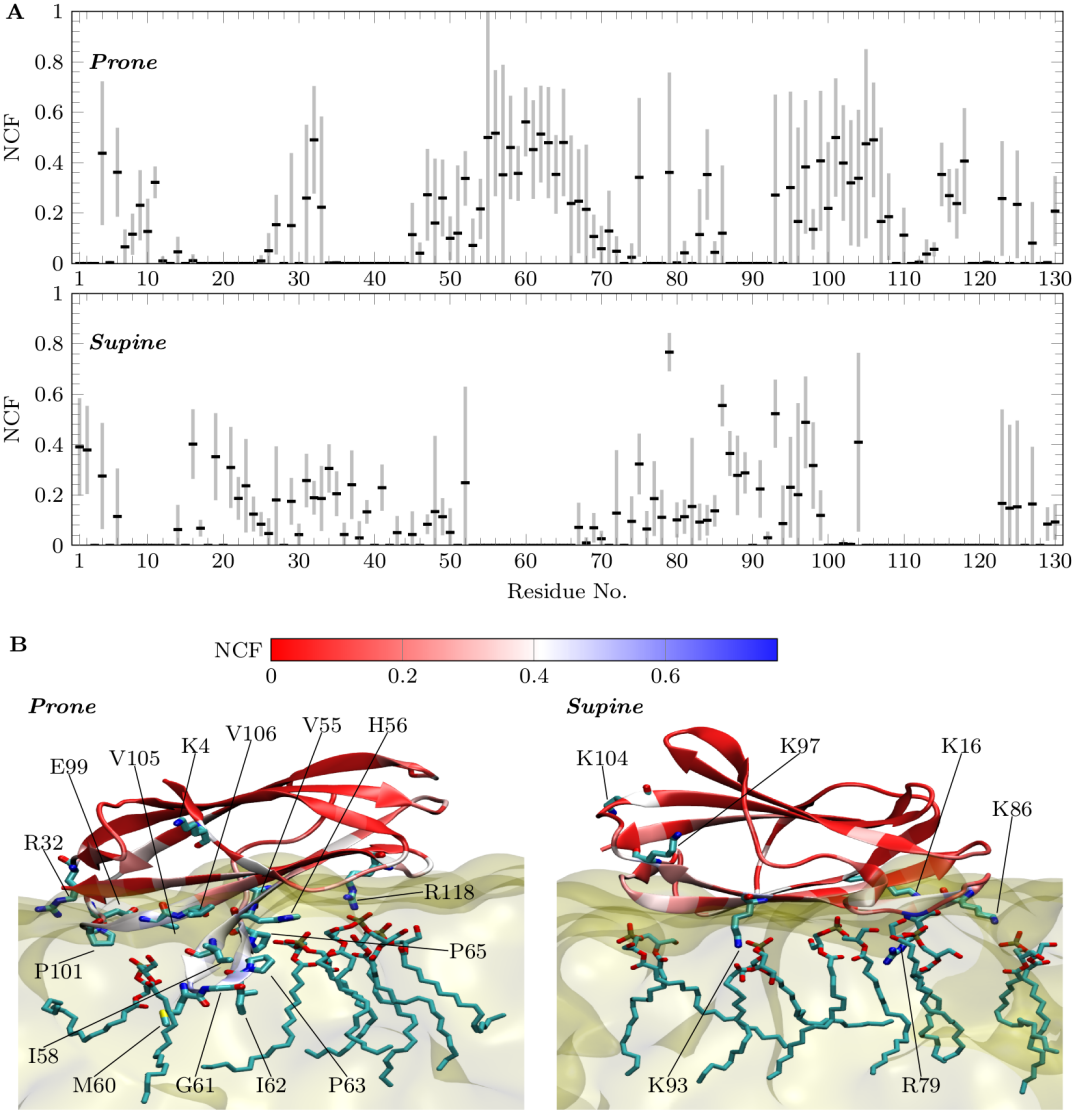
Normalized contact frequency analysis (ncf). A) The black rectangles show the ncf averaged over all bmp mixtures (bmp:chol:sm:dopg:popc = 20:10:0:0:70 (System 5), 20:35:0:0:45 (System 6) and 20:10:20:0:50 (System 7)), and the gray bars show the standard deviation. B) Representative snapshots showing npc2 interacting with bmp in *Prone mode* (left) and *Supine mode* (right). The protein is colored based on the ncf and the residues with ncf > 0.4 are shown in licorice and labeled.

Like all other neutral membranes, npc2 has small affinity for non-specific adsorption onto the sm-membrane surface (Δ*G* = −3 to −4 kJ/mol; Fig 4A). The bmp-sm membrane, on the other hand, has approximately the same affinity for npc2 when compared to bmp-membrane without sm but with 10 mol% chol (Δ*G* = −12 to −13 kJ/mol; Fig 4A). However, the important observation is that the deeper inserted binding mode of npc2^chol-bound^ (Fig 4A) is clearly inhibited by sm. Meanwhile, the deeper inserted state for npc2^*apo*^ has ∼4 kJ/mol higher free energy in all 20 mol% bmp membranes regardless of the presence of sm. Therefore, sm does not have any clear effect on membrane binding of npc2^*apo*^ (Fig 4A). These observations are also confirmed by comparing the 2d pmf data (Fig 4B) for sm-containing and sm-free membranes, as well as the lack of adsorption in *Supine mode* to sm-containing membranes in unbiased simulations (Table 1).

The results offer a mechanism as to how sm inhibits npc2-mediated cholesterol transport: sm can hinder cholesterol transfer between membranes by interfering with formation of the deeper inserted *Prone mode*, and thus, the cholesterol release from npc2^chol-bound^. Since bmp-sm membranes have similar affinity to those with only bmp, the sm-containing membrane fractions of the internal lysosomal/late endosomal membranes may arrest substantial amount of npc2 in non-conductive *Supine mode*. This can, in turn, reduce the effective concentration of npc2 available for cholesterol exchange, hindering it even further.

### Specific npc2-bmp interactions in *Prone mode* are based on the *membrane insertion loop*

Having identified two functional binding modes for npc2-membrane binding and the importance of bmp in the process, we moved on to characterize specific interactions between bmp and npc2 using all membrane-binding free energy simulations with bmp-membranes. To this end, for each orientation in each bmp-membrane free energy simulation, we calculated *the normalized contact frequency* (ncf) (S1 Text). For a given residue *r*, ncf describes the contact frequency with bmp compared to the total number of contacts with any lipids in a membrane. ncf captures if and how much bmp-npc2 interactions are enhanced with respect to those with other lipids in the membrane. In other words, for a particular residue, the values of ncf close to 1 indicate that its contact with bmp is likely specific, while values close to 0 indicate that either the contact was not observed or it is unspecific.

Fig 4A shows the average of ncf over all bmp-membrane free energy simulations, and its standard deviation. In *Prone mode*, the loop at the opening of the cholesterol-binding pocket (V59-M60-G61-I62-P63-V64-P65) along with several residues nearby in sequence has the highest probability of interacting with bmp when compared to any other lipid in the membrane (Fig 4A). Moreover, a stretch of residues from 96–108 that also line the bottom surface in *Prone mode*, as well as several residues around R118, are involved in bmp contacts. Likely due to the flexibility of the N-terminus, residues around K4 also form substantial contacts with bmp. Moreover, F66, V96, and T100, which have been previously implicated as “reversible gate keepers” in sterol uptake and release [18], are among the residues with enhanced specificity to bmp in *Prone mode*. This further supports the role of *Prone mode* and bmp in cholesterol transport.

Comparison of Fig 4A and 4B panels shows that *Supine mode* has considerably fewer specific contacts with bmp than *Prone mode*. The comparison also reveals that the residues in contact with bmp in the two orientations are anti-correlated except near the termini. This is partly the effect of the restricted definition of *Supine mode* used here. *Supine mode* appears to be more plastic than *Prone mode* and the low free energy region near *Supine mode* extends up to ∼100°.

While many polar and non-polar residues contact bmp in *Prone mode*, only positively charged residues interact with bmp in *Supine mode*. This is due to the deeper insertion taking place in *Prone mode*, which is stabilized by several nearby charged residue-bmp interactions (Fig 4). Moreover, H56, which is highly conserved among homologs of npc2, is likely involved in deep insertion of the loop into the membrane by interacting strongly with bmp. This residue is protonated in all of our simulations based on the p*K*_*a*_ estimations done on the crystal structures [3, 44]. Indeed, H56 may play a key role in the pH dependence of npc2-mediated cholesterol transport [27] by modulating the formation of deeply inserted *Prone mode*.

### Membrane lipids affect npc2-binding by altering the accessibility of bmp

Having established the role of bmp and its interaction with the *membrane insertion loop* for the formation of *Prone mode*, we also explored how the lipids studied here, in particular chol and sm, can affect this interaction at the molecular level. We showed that chol concentration differentially affects *Prone mode* in npc2^*apo*^ and npc2^chol-bound^, and sm hinders its formation. An important stabilizing factor for *Prone mode* is the anchoring by the *membrane insertion loop*, which is further stabilized by bmp. The insertion, on the other hand, is only favorable if the desolvation penalty of the loop and the cavity it inserts into are compensated by favorable interactions with lipids.

To indirectly assess the penalty of membrane insertion due to chol and sm, we use the quantity sasa-ratio = sasa_mil_/sasa_W_. Here, sasa_W_ stands for the solvent accessible surface area (sasa) estimated using a probe with a radius of 0.14 nm to approximate water as solvent; and sasa_mil_ stands for sasa estimated using a probe with a radius of 0.58 nm to approximate the *membrane insertion loop* (mil). The probe radius of 0.58 nm for mil is approximated by $R_{min}=0.066M^{\frac{1}{2}}$ [50], where mass, *M*, of mil is 694 Da. Note that *R*_*min*_ is the minimum radius of a sphere in which the protein of mass *M* can fit [50]. We calculated the sasa-ratio (reported as mean ± standard deviation) for all the lipid components collectively (sasa-ratio^membrane^) and for only bmp in the context of all lipid components (sasa-ratio^bmp^). For calculations, we used only free energy simulation trajectories (the last 50–100 ns), where the protein (npc2^*apo*^ or npc2^chol-bound^) is kept at a non-interacting distance to the membrane (*i* = 45–53, Fig 2) to ensure that membranes are unaffected by the protein.

sasa-ratio is a dimensionless quantity (within the interval of [0, 1]) that shows the relative accessibility of the surfaces by mil when compared to water, with values of ∼1 implying almost equal accessibility by mil and water, and values of ∼0 implying almost no accessibility by mil. The three studied systems (bmp:chol:sm:dopg:popc = 20:10:0:0:70 (System 5), 20:35:0:0:45 (System 6), and 20:10:20:0:50 (System 7)) have a similar sasa-ratio^membrane^ (the values being 0.53 ± 0.02, 0.51 ± 0.02, and 0.51 ± 0.02, respectively). This shows that the sasa-ratio does not depend substantially on the membrane size and composition, when all the membrane components are considered collectively. Interestingly, we found that increasing chol concentration from 10 to 35 mol % increased the sasa-ratio^bmp^ from 0.23 ± 0.06 to 0.32 ± 0.05. Meanwhile, inclusion of sm decreased the sasa-ratio^bmp^ to 0.20 ± 0.05. That is, chol enhances bmp presentation to mil, while sm decreases it.

Overall, this analysis suggests that bmp presentation through crevices on a membrane surface contributes strongly to formation of the deep-inserted *Prone mode*, and that bmp presentation is modulated by the relative concentrations of chol and sm. While chol elevates bmp presentation, sm has an opposite effect.

### Membrane-binding mode and membrane composition modulate the cholesterol-binding affinity of npc2

**Fig 5:**
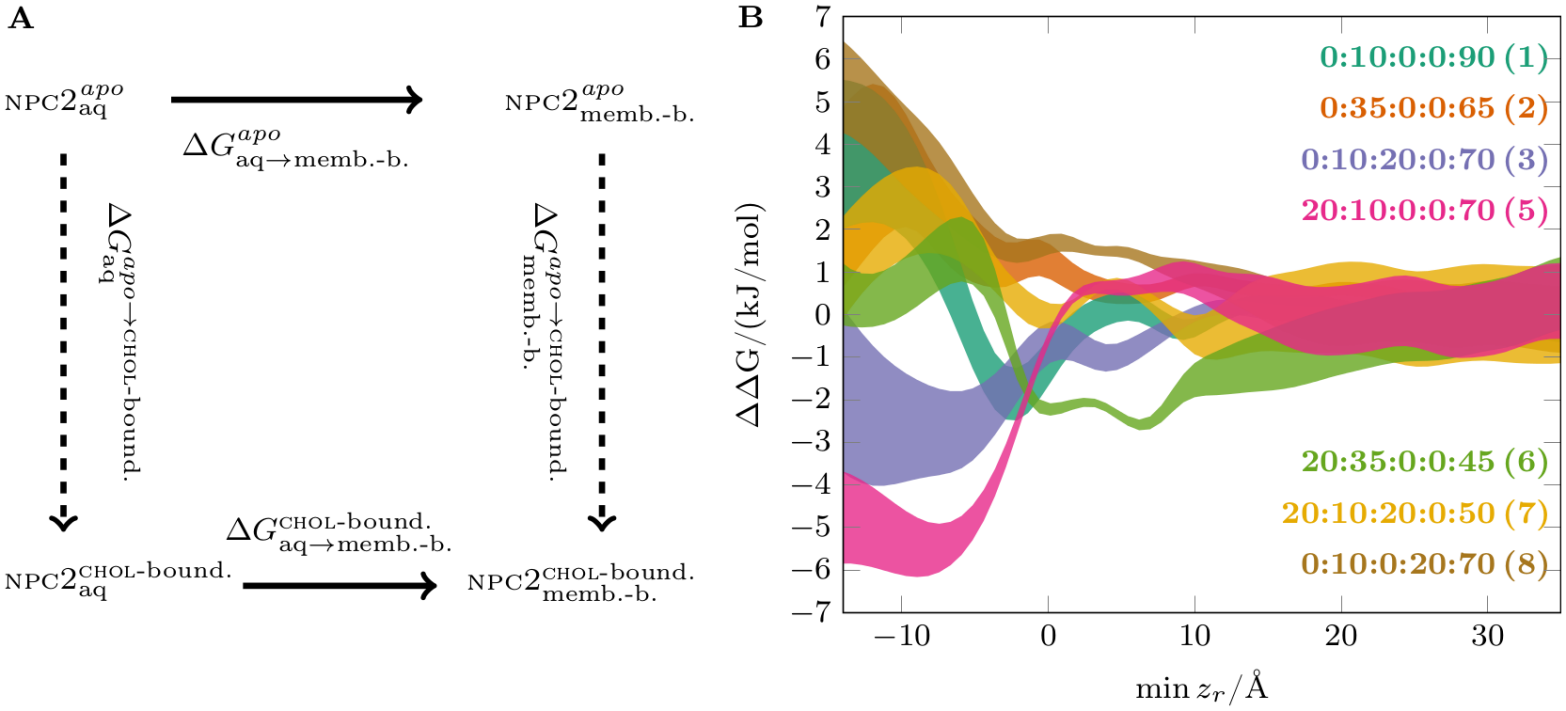
Cholesterol-binding free energy change upon membrane binding. A) Thermodynamic cycle for the estimation of cholesterol binding free energy change. Solid arrows represent the transformations performed in this work and dashed arrows represent the alternative transformations. B) The cholesterol binding free energy change as a function of min *z*_*r*_, calculated by ΔΔ*G*^chol-b.^(min *z_r_*) = Δ*G*^chol-b.^(min *z_r_*) − Δ*G*^*apo*^(min *z_r_*). The labels indicate membrane content in molar fractions for a mixture of bmp:chol:sm:dopg:popc, and the corresponding system numbers (Table 1) are provided in parentheses.

The thermodynamic cycle shown in Fig 5A allows us to infer about the cholesterol-binding free energy difference between the solvated (aqueous (aq)) and membrane-bound forms of npc2, which can be expressed as

$$\Delta \Delta G^{\text{CHOL}-\text{binding}}=\Delta G_{\text{membrane-bound}}^{apo\to \text{CHOL-bound}}-\Delta G_{\text{aq}}^{apo\to \text{CHOL-bound}}$$ (1)

$$=\Delta G_{\text{aq}\to \text{membrane-bound}}^{\text{CHOL}-\text{bound}}-\Delta G_{\text{aq}\to \text{membrane-bound}}^{apo},$$ (2)

where the apo → chol-bound and aq → membrane-bound transformations are shown with dashed and solid arrows, respectively, in Fig 5A. Expressing the aq → membrane-bound transformation in terms of min *z*_*r*_, we can calculate the relative cholesterol binding free energy, ΔΔ*G*^chol-bound^(min *z_r_*) (Fig 5B), by subtracting the pmf data for npc2^*apo*^ from those of npc2^chol-bound^ shown in Fig 3A.

The cholesterol binding affinity of npc2 is modulated by the composition of the membrane and the binding mode of npc2. For most of the studied membrane systems, the cholesterol binding free energy decreases upon formation of the deeply-inserted state of npc2 (Fig 5). However, for the low cholesterol bmp-membrane (bmp:chol:sm:dopg:popc = 20:10:0:0:70 (System 5)), the cholesterol binding affinity clearly increases up to ∼5 kJ/mol in the deeply-inserted membrane-bound state. The cholesterol-binding affinities are not affected in the shallow binding *Prone mode*, since the cholesterol-binding pocket is still exposed to bulk solvent. However, the deeply inserted state both changes the environment of the hydroxyl group of cholesterol by exposing it to the lipid head groups and likely affects the cholesterol-binding pocket.

In summary, membrane composition does not only determine the membrane binding properties of npc2, but also its cholesterol-binding affinity.

## Discussion

We performed an extensive set of atomistic simulations of the cholesterol-carrier protein npc2. The work done provides insights into the membrane-binding mechanism of npc2, its dependence on specific lipids, and the cholesterol transfer process associated with npc2.

**Fig 6:**
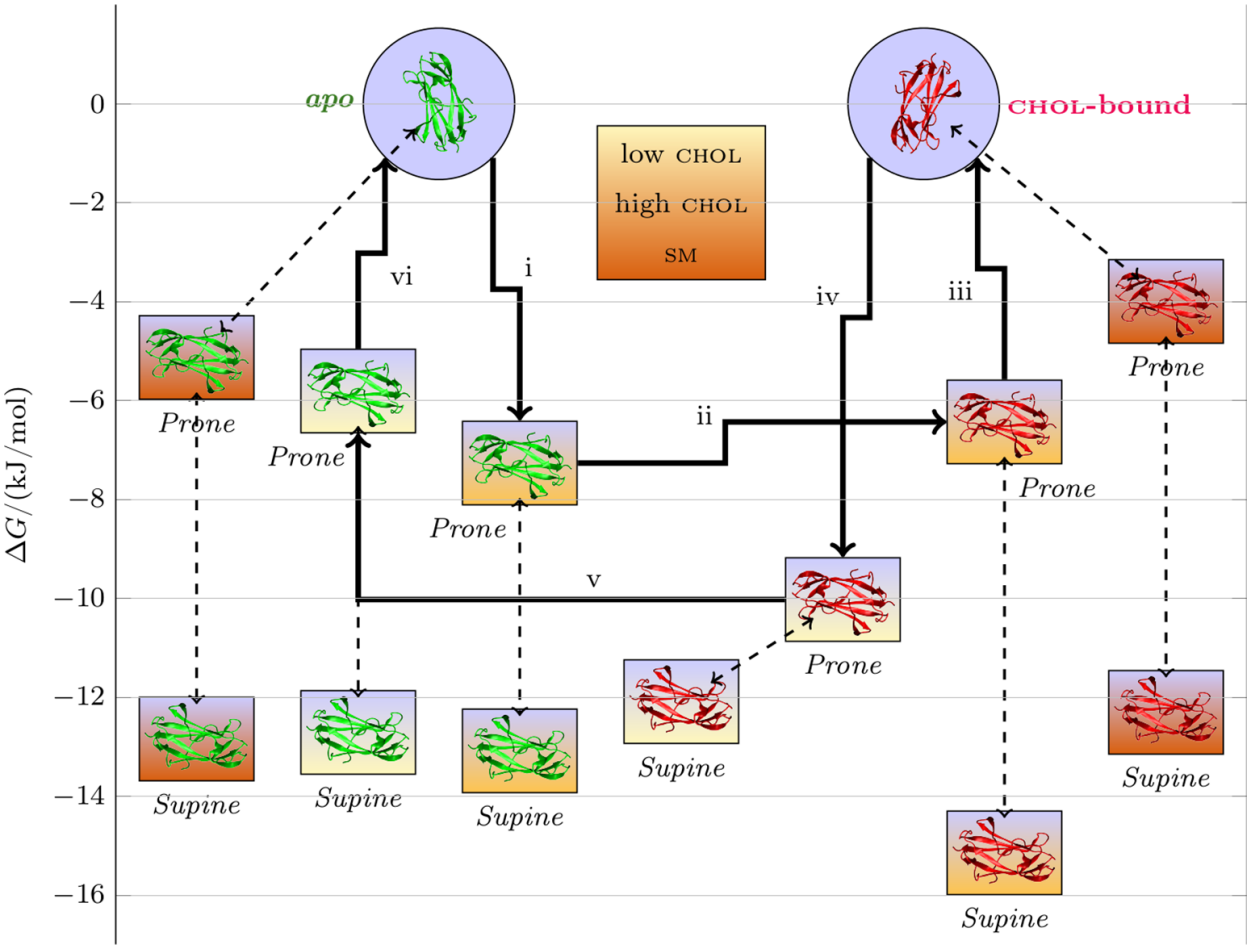
Schematic representation of the membrane binding of npc2. The thumbnail images for npc2^*apo*^ (green) and npc2^chol-bound^ (red) are placed on the *y*-axis based on the pmf data (Fig 3A) of the bmp:chol:sm:dopg:popc = 20:10:0:0:70 (System 5) (low chol), bmp:chol:sm:dopg:popc = 20:35:0:0:45 (System 6) (high chol), and bmp:chol:sm:dopg:popc = 20:10:20:0:50 (System 7) (low chol with sm) systems. A putative cycle for transferring cholesterol from high chol to low chol membranes are indicated by thick arrows. The competing processes (the interconversion between the two binding modes and binding to sm-containing membranes) are indicated by dashed arrows.

The results from the interactions between bmp-containing membranes and npc2 are summarized schematically in Fig 6. A simplified putative cycle for cholesterol transport between cholesterol-poor and cholesterol-rich membranes that contain bmp is represented by solid arrows. In this cycle, npc2^*apo*^ (i) binds a cholesterol-rich membrane in *Prone mode*, (ii) loads cholesterol, (iii) detaches from the membrane, (iv) binds a cholesterol-poor membrane in *Prone mode*, (v) unloads cholesterol, and (vi) detaches from the membrane. Based on this schematic, the interconversion between the *deeply-inserted* and the *surface-adsorbed* states, which have a free energy barrier of ∼6 − 7 kJ/mol depending on the membrane composition, competes with processes that are conductive to cholesterol exchange between membranes. npc2-sm membrane binding also competes with these processes by favoring the surface adsorbed states over the deeply inserted states.

We propose a concerted regulatory mechanism of npc2 by bmp and sm. In this mechanism, npc2-membrane binding is facilitated and stabilized by direct interaction of npc2 with bmp. While the negative charge of bmp is involved in pulling npc2 to the membrane surface, specific interactions, such as those with H56, are necessary especially for the formation of *Prone mode* and the insertion of the *membrane insertion loop*. Meanwhile, sm regulates membrane binding indirectly by modulating the presentation of bmp to npc2. Interestingly, this indirect regulation does not necessarily change the affinity of npc2 to the membrane, but instead alters its propensity to bind in *Prone mode*.

Our results have various implications about the mechanism and regulation of npc2-mediated cholesterol transport. Abdul-Hammed et al. have performed assays that showed bmp to enhance both processes, but sm to inhibit them [27]. Since the productive configuration for cholesterol exchange between the membranes and npc2 is likely to be *Prone mode*, our results explain the underlying molecular mechanism for the roles of bmp and sm. Essentially, bmp supports npc2-mediated cholesterol transport by enhancing binding in *Prone mode*, and sm in turn inhibits it by the reverse mechanism. Besides, acid sphingomyelinase regulates npc2-mediated cholesterol transport by converting sm to ceramide [51]. The functional role of these two different binding orientations in cholesterol transport can be explained by this regulation mechanism. That is, cholesterol transport rate can be controlled by the composition of the membrane interacting with npc2 such that npc2-binding in *Prone mode* is favored, or disfavored. Another functional role for two distinct high affinity surfaces of the protein is potentially related to npc2’s fusogenic function and its regulation [27].

npc2 contains many acidic and basic residues, mainly on its surface. The bovine and human orthologs of the protein have a net charge of +2*e* and +1*e*, respectively, based on the number of charged residues. In the low pH of lysosomes, at least some acidic residues and histidines are expected to be protonated attaining neutral or positive charges and increasing the overall positive charge of the protein. Indeed, in our simulations, we used bovine npc2 protonated at E110 (Q in human), H31 (S in human) and H56 (strongly conserved) based on p*K*_a_ estimations on the crystal structures to approximate an environmental pH of ∼5. However, our simulations do not account for dynamic shifts in p*K*_a_ due to changes in the local environment. Several acidic residues are located on the membrane interacting surfaces in *Prone mode* and *Supine mode*. When npc2 adsorbs onto the membrane surface, the local electrostatic potential due to the anionic lipid head groups will increase the intrinsic p*K*_a_ of these residues. That is, some acidic residues can get protonated depending on the membrane composition and depending on where they are located with respect to the membrane in a particular membrane binding mode. This can, in turn, alter membrane binding modes and their affinities, especially in the anionic membranes. For example, D113, which is on the membrane-interacting surface in *Prone mode*, has been shown to be vital for cholesterol transport, but not for cholesterol binding [24]. The dynamic and differential protonation of residues like D113 may modulate npc2-binding to membranes with different compositions. Besides, they likely prevent very tight npc2-membrane binding. This is essential for efficient shuttling of npc2 between disconnected membranes to transport cholesterol.

Glycosylation of npc2 has been implicated in proper protein sorting, protection against degradation by the lysosomal enzymes, and in modulating cholesterol transfer rates [26]. Monoglycosylated (glycosylated at N39) and diglycosylated (glycosylated at both N39 and N116) forms of npc2 have been identified in lysosomes [26, 52]. Cholesterol transfer by the diglycosylated form has been shown to be slower than that by the monoglycosylated form [26], but the molecular mechanism accounting for the observed difference remains unknown. Based on our work, N39 and N116 are located on the membrane interacting surfaces in *Supine mode* and *Prone mode*, respectively. Glycosylation of N39 may destabilize *Supine mode* and shift the equilibrium towards *Prone mode*. This shift, in turn, may enhance cholesterol uptake and release, since this process presumably takes place in *Prone mode*, but not in *Supine mode*. On the other hand, glycosylation at N116 is likely to interfere with the membrane interactions in *Prone mode*, especially close to the C-terminal side of the protein (Fig 4A). Cholesterol uptake and release could still take place, albeit slower, because the stretch of residues in the proximity of the hydrophobic loop may still anchor the protein to the membrane.

In summary, our results provide an atom-scale mechanism that explains the differential regulatory roles of bmp and sm in npc2-membrane binding. npc2 binds a membrane in two different binding orientations depending on the lipid composition. bmp is required for npc2-membrane binding and cannot be substituted by other anionic lipids. On the other hand, sm counteracts bmp and hinders the formation of the deep-inserted mode that places the cholesterol-binding pocket in direct contact with the membrane surface and is conductive for cholesterol uptake/release.

## Supporting information

S1 Text Supplementary text. Detailed descriptions of bmp parametrization, simulation system preparation, free energy calculations and reweighting approach are given.
(PDF)

S1 Fig The free energy surfaces for npc2 binding to membranes as a function of |*z*|. The free energy surfaces for cholesterol-bound (left column) and *apo* (right column) npc2 binding to membranes with different compositions. A) Potential of mean force (pmf) profiles shown as a function of |*z*|. The band thickness displays the error. B) pmf surfaces shown as a function of |*z*| and *ϕ*. Contours were plotted every 2 kJ/mol increments. The two binding orientations are marked with dashed lines. The thumbnail images of npc2 structure for the corresponding orientations are shown on the upper left for *Prone mode* and upper right for *Supine mode*. The labels indicate membrane content in molar fractions for a mixture of bmp:chol:sm:dopg:popc, and the corresponding system numbers (Table 1) are provided in parentheses. The local errors are given in S2 Fig.
(TIFF)

S2 Fig Local errors of |*z*| vs. *ϕ* free energy surfaces for anionic membranes. Local errors of |*z*| vs. *ϕ* free energy surfaces for cholesterol-bound (left column) and *apo* (right column) npc2 binding to anionic membranes with indicated compositions. The two binding orientations are marked with dashed lines. The labels indicate membrane content in molar fractions for a mixture of bmp:chol:sm:dopg:popc, and the corresponding system numbers (Table 1) are provided in parentheses.
(TIFF)

S3 Fig The |*z*| vs. *ϕ* free energy surfaces for neutral membranes. The |*z*| vs. *ϕ* free energy surfaces for cholesterol-bound (left column) and *apo* (right column) npc2 binding to neutral membranes with indicated compositions. The two binding orientations are marked with dashed lines. The labels indicate membrane content in molar fractions for a mixture of bmp:chol:sm:dopg:popc, and the corresponding system numbers (Table 1) are provided in parentheses. The local errors are given in S4 Fig.
(TIFF)

S4 Fig Local errors of |*z*| vs. *ϕ* free energy surfaces for neutral membranes. Local errors of |*z*| vs. *ϕ* free energy surfaces for cholesterol-bound (left column) and *apo* (right column) npc2 binding to neutral membranes with indicated compositions. The two binding orientations are marked with dashed lines. The labels indicate membrane content in molar fractions for a mixture of bmp:chol:sm:dopg:popc, and the corresponding system numbers (Table 1) are provided in parentheses.
(TIFF)

S5 Fig The |*z*| vs. *θ* free energy surfaces for anionic membranes. The |*z*| vs. *θ* free energy surfaces for cholesterol-bound (left column) and *apo* (right column) npc2 binding to anionic membranes with indicated compositions. The labels indicate membrane content in molar fractions for a mixture of bmp:chol:sm:dopg:popc, and the corresponding system numbers (Table 1) are provided in parentheses. The local errors are given in S6 Fig.
(TIFF)

S6 Fig Local errors of |*z*| vs. *θ* free energy surfaces for anionic membranes. Local errors of |*z*| vs. *θ* free energy surfaces for cholesterol-bound (left column) and *apo* (right column) npc2 binding to anionic membranes with indicated compositions. The labels indicate membrane content in molar fractions for a mixture of bmp:chol:sm:dopg:popc, and the corresponding system numbers (Table 1) are provided in parentheses.
(TIFF)

S7 Fig The |*z*| vs. *θ* free energy surfaces for neutral membranes. The |*z*| vs. *θ* free energy surfaces for cholesterol-bound (left column) and *apo* (right column) npc2 binding to neutral membranes with indicated compositions. The labels indicate membrane content in molar fractions for a mixture of bmp:chol:sm:dopg:popc, and the corresponding system numbers (Table 1) are provided in parentheses. The local errors are given in S8 Fig.
(TIFF)

S8 Fig Local errors of |*z*| vs. *θ* free energy surfaces for neutral membranes. Local errors of |*z*| vs. *θ* free energy surfaces for cholesterol-bound (left column) and *apo* (right column) npc2 binding to neutral membranes with indicated compositions. The labels indicate membrane content in molar fractions for a mixture of bmp:chol:sm:dopg:popc, and the corresponding system numbers (Table 1) are provided in parentheses.
(TIFF)

S9 Fig Local errors of min *z*_*r*_ vs. *ϕ* free energy surfaces. Local errors of min *z*_*r*_ vs. *ϕ* free energy surfaces for cholesterol-bound (left column) and *apo* (right column) npc2 binding to charged membranes with indicated compositions. The two binding orientations are marked with dashed lines. The labels indicate membrane content in molar fractions for a mixture of bmp:chol:sm:dopg:popc, and the corresponding system numbers (Table 1) are provided in parentheses.
(TIFF)

S10 Fig The min *z*_*r*_ vs. *ϕ* free energy surfaces for neutral membranes. The min *z*_*r*_ vs. *ϕ* free energy surfaces for cholesterol-bound (left column) and *apo* (right column) npc2 binding to neutral membranes with the indicated compositions. The two binding orientations are marked with dashed lines. The labels indicate membrane content in molar fractions for a mixture of bmp:chol:sm:dopg:popc, and the corresponding system numbers (Table 1) are provided in parentheses. The local errors are given in S11 Fig.
(TIFF)

S11 Fig Local errors of min *z*_*r*_ vs. *ϕ* free energy surfaces for neutral membranes. Local errors of min *z*_*r*_ vs. *ϕ* free energy surfaces for cholesterol-bound (left column) and *apo* (right column) npc2 binding to neutral membranes with indicated compositions. The two binding orientations are marked with dashed lines. The labels indicate membrane content in molar fractions for a mixture of bmp:chol:sm:dopg:popc, and the corresponding system numbers (Table 1) are provided in parentheses.
(TIFF)

S12 Fig Electrostatic potential profiles. Electrostatic potential profiles for the neutral (A) and charged (B) membranes. The profiles are calculated from free energy simulation trajectories (the last 100 ns), where the protein (npc2^*apo*^) is kept at a non-interacting distance to the membrane (*i* = 45–53, Fig 2) to ensure that membranes are unaffected by the protein. The trajectories were first centered based membrane com and then, the electric field calculated by gmx potential tool included in gromacs 5.0 [29] is averaged over the upper and lower halves of the box for symmetry. The electric field is then integrated to get the electrostatic potential profiles for each simulation. The profiles were averaged over all relevant simulations of a particular system and the band thickness displays their standard error. The location of the membrane P atoms is indicated with gray bands.
(TIFF)

## References

[1] Ikonen E. Cellular Cholesterol Trafficking and Compartmentalization. Nat Rev Mol Cell Biol. 2008;9(2):125–138. doi: 10.1038/nrm2336 18216769 10.1038/nrm2336

[2] Phillips MC. Molecular Mechanisms of Cellular Cholesterol Efflux. J Biol Chem. 2014;289(35):24020–24029. doi: 10.1074/jbc.R114.583658 25074931 10.1074/jbc.R114.583658 PMC4148835

[3] Xu S, Benoff B, Liou HL, Lobel P, Stock AM. Structural Basis of Sterol Binding by NPC2, a Lysosomal Protein Deficient in Niemann-Pick Type C2 Disease. J Biol Chem. 2007;282(32):23525–23531. doi: 10.1074/jbc.M703848200 17573352 10.1074/jbc.M703848200 PMC3645284

[4] Subramanian K, Balch WE. NPC1/NPC2 Function as a Tag Team Duo to Mobilize Cholesterol. Proc Natl Acad Sci USA. 2008;105(40):15223–15224. doi: 10.1073/pnas.0808256105 18832164 10.1073/pnas.0808256105 PMC2563125

[5] Infante RE, Wang ML, Radhakrishnan A, Kwon HJ, Brown MS, Goldstein JL. NPC2 Facilitates Bidirectional Transfer of Cholesterol between NPC1 and Lipid Bilayers, a Step in Cholesterol Egress from Lysosomes. Proc Natl Acad Sci USA. 2008;105(40):15287–15292. doi: 10.1073/pnas.0807328105 18772377 10.1073/pnas.0807328105 PMC2563079

[6] Bi X, Liao G. Cholesterol in Niemann–Pick Type C Disease In: Harris JR, editor. Cholesterol Binding and Cholesterol Transport Proteins:. vol. 51 Dordrecht: Springer Netherlands; 2010 p. 319–335. 10.1007/978-90-481-8622-8_11 PMC4212451 20213549

[7] Chang TY, Reid PC, Sugii S, Ohgami N, Cruz JC, Chang CCY. Niemann-Pick Type C Disease and Intracellular Cholesterol Trafficking. J Biol Chem. 2005;280(22):20917–20920. doi: 10.1074/jbc.R400040200 15831488 10.1074/jbc.R400040200

[8] Dixit SS, Jadot M, Sohar I, Sleat DE, Stock AM, Lobel P. Loss of Niemann-Pick C1 or C2 Protein Results in Similar Biochemical Changes Suggesting That These Proteins Function in a Common Lysosomal Pathway. PLoS ONE. 2011;6(8):e23677 doi: 10.1371/journal.pone.0023677 21887293 10.1371/journal.pone.0023677 PMC3161064

[9] Wang ML, Motamed M, Infante RE, Abi-Mosleh L, Kwon HJ, Brown MS, et al Identification of Surface Residues on Niemann-Pick C2 Essential for Hydrophobic Handoff of Cholesterol to NPC1 in Lysosomes. Cell Metab. 2010;12(2):166–173. doi: 10.1016/j.cmet.2010.05.016 20674861 10.1016/j.cmet.2010.05.016 PMC3034247

[10] Deffieu MS, Pfeffer SR. Niemann-Pick Type C 1 Function Requires Lumenal Domain Residues That Mediate Cholesterol-Dependent NPC2 Binding. Proc Natl Acad Sci. 2011;108(47):18932–18936. doi: 10.1073/pnas.1110439108 22065762 10.1073/pnas.1110439108 PMC3223457

[11] Gong X, Qian H, Zhou X, Wu J, Wan T, Cao P, et al Structural Insights into the Niemann-Pick C1 (NPC1)-Mediated Cholesterol Transfer and Ebola Infection. Cell. 2016;165(6):1467–1478. doi: 10.1016/j.cell.2016.05.022 27238017 10.1016/j.cell.2016.05.022 PMC7111323

[12] Li X, Saha P, Li J, Blobel G, Pfeffer SR. Clues to the Mechanism of Cholesterol Transfer from the Structure of NPC1 Middle Lumenal Domain Bound to NPC2. Proc Natl Acad Sci. 2016; p. 201611956. 10.1073/pnas.1611956113 PMC5018801 27551080

[13] Li X, Wang J, Coutavas E, Shi H, Hao Q, Blobel G. Structure of Human Niemann–Pick C1 Protein. Proc Natl Acad Sci. 2016;113(29):8212–8217. doi: 10.1073/pnas.1607795113 27307437 10.1073/pnas.1607795113 PMC4961162

[14] Zhao Y, Ren J, Harlos K, Stuart DI. Structure of Glycosylated NPC1 Luminal Domain C Reveals Insights into NPC2 and Ebola Virus Interactions. FEBS Lett. 2016;590(5):605–612. doi: 10.1002/1873-3468.12089 26846330 10.1002/1873-3468.12089 PMC4819692

[15] Kwon HJ, Abi-Mosleh L, Wang ML, Deisenhofer J, Goldstein JL, Brown MS, et al Structure of N-Terminal Domain of NPC1 Reveals Distinct Subdomains for Binding and Transfer of Cholesterol. Cell. 2009;137(7):1213–1224. doi: 10.1016/j.cell.2009.03.049 19563754 10.1016/j.cell.2009.03.049 PMC2739658

[16] Estiu G, Khatri N, Wiest O. Computational Studies of the Cholesterol Transport between NPC2 and the N-Terminal Domain of NPC1 (NPC1(NTD)). Biochemistry (Mosc). 2013;52(39):6879–6891. doi: 10.1021/bi4005478 10.1021/bi4005478 24001314

[17] Elghobashi-Meinhardt N. Niemann–Pick Type C Disease: A QM/MM Study of Conformational Changes in Cholesterol in the NPC1(NTD) and NPC2 Binding Pockets. Biochemistry (Mosc). 2014;53(41):6603–6614. doi: 10.1021/bi500548f 10.1021/bi500548f 25251378

[18] Poongavanam V, Kongsted J, Wüstner D. Computational Analysis of Sterol Ligand Specificity of the Niemann Pick C2 Protein. Biochemistry. 2016;55(36):5165–5179. doi: 10.1021/acs.biochem.6b00217 27533706 10.1021/acs.biochem.6b00217

[19] Storch J, Xu Z. Niemann–Pick C2 (NPC2) and Intracellular Cholesterol Trafficking. Biochim Biophys Acta BBA—Mol Cell Biol Lipids. 2009;1791(7):671–678. doi: 10.1016/j.bbalip.2009.02.001 10.1016/j.bbalip.2009.02.001 PMC4281484 19232397

[20] Boadu E, Nelson RC, Francis GA. ABCA1-Dependent Mobilization of Lysosomal Cholesterol Requires Functional Niemann–Pick C2 but Not Niemann–Pick C1 Protein. Biochim Biophys Acta BBA—Mol Cell Biol Lipids. 2012;1821(3):396–404. doi: 10.1016/j.bbalip.2011.11.013 10.1016/j.bbalip.2011.11.013 22179027

[21] Xu Z, Farver W, Kodukula S, Storch J. Regulation of Sterol Transport between Membranes and NPC2. Biochemistry. 2008;47(42):11134–11143. doi: 10.1021/bi801328u 18823126 10.1021/bi801328u PMC4355403

[22] Kobayashi T, Beuchat MH, Chevallier J, Makino A, Mayran N, Escola JM, et al Separation and Characterization of Late Endosomal Membrane Domains. J Biol Chem. 2002;277(35):32157–32164. doi: 10.1074/jbc.M202838200 12065580 10.1074/jbc.M202838200

[23] Schulze H, Sandhoff K. Sphingolipids and Lysosomal Pathologies. Biochim Biophys Acta BBA—Mol Cell Biol Lipids. 2014;1841(5):799–810. doi: 10.1016/j.bbalip.2013.10.015 10.1016/j.bbalip.2013.10.015 24184515

[24] McCauliff LA, Xu Z, Li R, Kodukula S, Ko DC, Scott MP, et al Multiple Surface Regions on the Niemann-Pick C2 Protein Facilitate Intracellular Cholesterol Transport. J Biol Chem. 2015;290(45):27321–27331. doi: 10.1074/jbc.M115.667469 26296895 10.1074/jbc.M115.667469 PMC4646368

[25] Jochum A, Li R, Newton S, McCauliff L, Storch J. The Unique Relationship between Niemann Pick Type C2 (NPC2) Protein and Lysobisphosphatidic Acid (LBPA). FASEB J. 2016;30(1 Supplement):658.2.

[26] Cheruku SR, Xu Z, Dutia R, Lobel P, Storch J. Mechanism of Cholesterol Transfer from the Niemann-Pick Type C2 Protein to Model Membranes Supports a Role in Lysosomal Cholesterol Transport. J Biol Chem. 2006;281(42):31594–31604. doi: 10.1074/jbc.M602765200 16606609 10.1074/jbc.M602765200

[27] Abdul-Hammed M, Breiden B, Adebayo MA, Babalola JO, Schwarzmann G, Sandhoff K. Role of Endosomal Membrane Lipids and NPC2 in Cholesterol Transfer and Membrane Fusion. J Lipid Res. 2010;51(7):1747–1760. doi: 10.1194/jlr.M003822 20179319 10.1194/jlr.M003822 PMC2882726

[28] Barducci A, Bussi G, Parrinello M. Well-Tempered Metadynamics: A Smoothly Converging and Tunable Free-Energy Method. Phys Rev Lett. 2008;100(2). doi: 10.1103/PhysRevLett.100.020603 18232845 10.1103/PhysRevLett.100.020603

[29] Abraham MJ, Murtola T, Schulz R, Páll S, Smith JC, Hess B, et al GROMACS: High Performance Molecular Simulations through Multi-Level Parallelism from Laptops to Supercomputers. SoftwareX. 2015;1-2:19–25. doi: 10.1016/j.softx.2015.06.001

[30] Lindorff-Larsen K, Piana S, Palmo K, Maragakis P, Klepeis JL, Dror RO, et al Improved Side-Chain Torsion Potentials for the Amber ff99SB Protein Force Field. Proteins. 2010;78(8):1950–1958. doi: 10.1002/prot.22711 20408171 10.1002/prot.22711 PMC2970904

[31] Jämbeck JPM, Lyubartsev AP. Another Piece of the Membrane Puzzle: Extending Slipids Further. J Chem Theory Comput. 2013;9(1):774–784. doi: 10.1021/ct300777p 26589070 10.1021/ct300777p

[32] Jorgensen WL, Chandrasekhar J, Madura JD, Impey RW, Klein ML. Comparison of Simple Potential Functions for Simulating Liquid Water. J Chem Phys. 1983;79(2):926–935. doi: 10.1063/1.445869

[33] Hess B, Bekker H, Berendsen HJC, Fraaije JGEM. LINCS: A Linear Constraint Solver for Molecular Simulations. J Comput Chem. 1997;18(12):1463–1472. doi: 10.1002/(SICI)1096-987X(199709)18:12%3C1463::AID-JCC4%3E3.0.CO;2-H

[34] Essmann U, Perera L, Berkowitz ML, Darden T, Lee H, Pedersen LG. A Smooth Particle Mesh Ewald Method. J Chem Phys. 1995;103(19):8577–8593. doi: 10.1063/1.470117

[35] Darden T, York D, Pedersen L. Particle Mesh Ewald: An N⋅log(N) Method for Ewald Sums in Large Systems. J Chem Phys. 1993;98(12):10089–10092. doi: 10.1063/1.464397

[36] Allen MP, Tildesley DJ. Computer Simulation of Liquids. New York, NY, USA: Clarendon Press; 1989.

[37] Bussi G, Donadio D, Parrinello M. Canonical Sampling through Velocity Rescaling. J Chem Phys. 2007;126(1):014101 doi: 10.1063/1.2408420 17212484 10.1063/1.2408420

[38] Berendsen HJC, Postma JPM, van Gunsteren WF, DiNola A, Haak JR. Molecular Dynamics with Coupling to an External Bath. J Chem Phys. 1984;81(8):3684–3690. doi: 10.1063/1.448118

[39] Nosé S. A Molecular Dynamics Method for Simulations in the Canonical Ensemble. Mol Phys. 1984;52(2):255–268. doi: 10.1080/00268978400101201

[40] Hoover WG. Canonical Dynamics: Equilibrium Phase-Space Distributions. Phys Rev A. 1985;31(3):1695–1697. doi: 10.1103/PhysRevA.31.1695 10.1103/physreva.31.1695 9895674

[41] Parrinello M, Rahman A. Polymorphic Transitions in Single Crystals: A New Molecular Dynamics Method. J Appl Phys. 1981;52(12):7182–7190. doi: 10.1063/1.328693

[42] Nosé S, Klein ML. Constant Pressure Molecular Dynamics for Molecular Systems. Mol Phys. 1983;50(5):1055–1076. doi: 10.1080/00268978300102851

[43] Tribello GA, Bonomi M, Branduardi D, Camilloni C, Bussi G. PLUMED 2: New Feathers for an Old Bird. Computer Physics Communications. 2014;185(2):604–613. doi: 10.1016/j.cpc.2013.09.018

[44] Friedland N, Liou HL, Lobel P, Stock AM. Structure of a Cholesterol-Binding Protein Deficient in Niemann-Pick Type C2 Disease. Proc Natl Acad Sci USA. 2003;100(5):2512–2517. doi: 10.1073/pnas.0437840100 12591954 10.1073/pnas.0437840100 PMC151372

[45] Marinelli F, Pietrucci F, Laio A, Piana S. A Kinetic Model of Trp-Cage Folding from Multiple Biased Molecular Dynamics Simulations. PLoS Comput Biol. 2009;5(8):e1000452 doi: 10.1371/journal.pcbi.1000452 19662155 10.1371/journal.pcbi.1000452 PMC2711228

[46] Johnston JM, Wang H, Provasi D, Filizola M. Assessing the Relative Stability of Dimer Interfaces in G Protein-Coupled Receptors. PLoS Comput Biol. 2012;8(8):e1002649 doi: 10.1371/journal.pcbi.1002649 22916005 10.1371/journal.pcbi.1002649 PMC3420924

[47] Tiwary P, Parrinello M. A Time-Independent Free Energy Estimator for Metadynamics. J Phys Chem B. 2015;119(3):736–742. doi: 10.1021/jp504920s 25046020 10.1021/jp504920s

[48] Bartels C. Analyzing Biased Monte Carlo and Molecular Dynamics Simulations. Chem Phys Lett. 2000;331(5-6):446–454. doi: 10.1016/S0009-2614(00)01215-X

[49] Bissig C, Gruenberg J. Lipid Sorting and Multivesicular Endosome Biogenesis. Cold Spring Harb Perspect Biol. 2013;5(10):a016816–a016816. doi: 10.1101/cshperspect.a016816 24086044 10.1101/cshperspect.a016816 PMC3783046

[50] Erickson HP. Size and Shape of Protein Molecules at the Nanometer Level Determined by Sedimentation, Gel Filtration, and Electron Microscopy. Biol Proced Online. 2009;11(1):32–51. doi: 10.1007/s12575-009-9008-x 19495910 10.1007/s12575-009-9008-x PMC3055910

[51] Gallala HD, Breiden B, Sandhoff K. Regulation of the NPC2 Protein-Mediated Cholesterol Trafficking by Membrane Lipids. J Neurochem. 2011;116(5):702–707. doi: 10.1111/j.1471-4159.2010.07014.x 21214551 10.1111/j.1471-4159.2010.07014.x

[52] Liou HL, Dixit SS, Xu S, Tint GS, Stock AM, Lobel P. NPC2, the Protein Deficient in Niemann-Pick C2 Disease, Consists of Multiple Glycoforms That Bind a Variety of Sterols. J Biol Chem. 2006;281(48):36710–36723. doi: 10.1074/jbc.M608743200 17018531 10.1074/jbc.M608743200

