# Supplementary material for: Concerted regulation of npc2 binding to endosomal/lysosomal membranes by bis(monoacylglycero)phosphate and sphingomyelin
Source: PLoS Comput Biol. 2017 Oct 30;13(10):e1005831. doi: 10.1371/journal.pcbi.1005831 (PMC5679659; doi:10.1371/journal.pcbi.1005831)
Supplement: S1 Text — Detailed descriptions of bmp parametrization, simulation system preparation, free energy calculations and reweighting approach are given. (PDF) [file pcbi.1005831.s001.pdf]

Supplementary text:
Concerted regulation of NPC2 binding to endosomal/lysosomal membranes by bis(monoacylglycero)phosphate and sphingomyelin

Giray Enkavi[1,2], Heikki Mikkolainen[1], Burçin Güngör[3,4], Elina Ikonen[3,4], Ilpo Vattulainen[1,2,5*]

1 Department of Physics, Tampere University of Technology, FI-33101 Tampere, Finland

2 Department of Physics, P.O. Box 64, FI-00014 University of Helsinki, Finland

3 Department of Anatomy, Faculty of Medicine, P.O. Box 63, FI-00014 University of Helsinki, Finland

4 Minerva Foundation Institute for Medical Research, Helsinki, Finland

5 Memphys–Center for Biomembrane Physics, University of Southern Denmark, Odense, Denmark

* ilpo.vattulainen@helsinki.fi

S1 Materials and methods

S1.1 Parametrization of BMP

We initially built 2,2'- and 3,3'-dioleoyl lysobisphosphatidic acid (2,2'-BMP18:1 and 3,3'-BMP18:1) topologies based on the CHARMM36 additive force field for lipids [1] using analogy. That is, we adopted all charges and atom types from other 18:1 lipids already included in the CHARMM36 force field [1]. Then, we used CHARMM-GUI [2] to generate a bilayer of 128 DOPG lipids and mutated each DOPG to 2,2'-BMP18:1 and 3,3'-BMP18:1 separately to construct two different bilayers composed of 128 copies of 2,2'-BMP18:1 and 128 copies of 3,3'-BMP18:1. We performed minimization followed by a series of equilibration steps, in which we gradually removed the restraints as suggested by CHARMM-GUI [2] using GROMACS 5.0 [3]. We finally simulated the bilayer without restraints for 100 ns in NpT at 310 K and 1 atm pressure.

To derive atomic charges that are consistent with the Slipids force field [4], we followed the Slipids charge parametrization scheme. 52 random BMP conformations from the last ∼25 ns of the simulation performed using the CHARMM36 force field [1] were selected. Head groups and the glycerol region including the ester groups of each 52 lipids were first geometry optimized. Then, the partial atomic charges were computed with the multiple conformations and orientations restrained electrostatic potential (RESP) approach [5] using three different orientations for each molecular fragment. In the RESP calculations, charges of the ester group atoms were constrained to the values already present in the Slipids force field [4].

All quantum-chemical calculations were performed using the Gaussian09 program suite [6] at the B3LYP/cc-pVTZ level of theory. Charge calculations were performed with the IEFPCM model

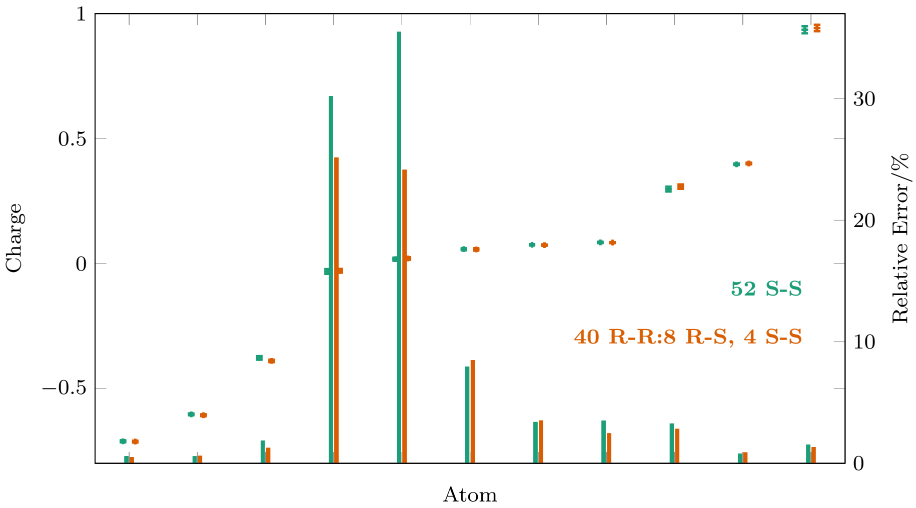

Fig s1. Comparison of results of charge calculations performed on the head groups and the glycerol region of 2,2'-BMP18:1 S-S'. Charges were calculated using multiple conformations and orientations RESP fitting using 52 random head group conformations picked from a uniformly S-S BMP or R-R, R-S, and S-S mixture BMP membranes. Error estimations were performed by generating 100 bootstrap samples from the 52 head group conformations and refitting the charges. (Left axis) Charge and their statistical error for each unique atom is shown. (Right axis) The relative errors, means divided by the statistical error, (spikes) are also shown.

in a polarizable continuum with a dielectric constant of 78.4 by setting solvent to water. Multiple conformations and orientations RESP fitting was performed with the R.E.D. III.5 software [5].

The BMP can exist as R-R, S-S, and R-S stereoisomers. We tested for the effect of stereoisomerization for charge calculations by performing the above protocol for 2,2'-BMP18:1 head groups conformations that are uniformly S-S, and also for R-R, R-S, and S-S mixtures. The estimated charges were found to be the same indicating that this stereoisomerization does not effect the charge estimation using the above protocol (Fig s1). Since S-S 2,2'-BMP is the most biologically active form and interacts with NPC2 most favorably [7], we used this configuration in all subsequent simulations.

s1.2 Preparation of simulation systems and unbiased simulations

We prepared and equilibrated membranes and proteins separately before combining them to use in the biased and unbiased protein-membrane simulations. All bilayers listed in Table 1 were prepared in 0.1 M NaCl solution using CHARMM-GUI [2]. The systems with 10 mol% cholesterol contain a total of 160 lipids, while those with 35 mol% cholesterol contain 200 lipids, so as to have a sufficiently large membrane area for protein-membrane binding simulations. BMP-containing systems were prepared by replacing the head groups of DOPG-containing bilayers built using CHARMM-GUI [2]. We minimized the BMP-containing systems in several stages, checking and correcting the stereochemistry of head groups at each stage. For all membranes, we first performed the staged minimization and equilibration protocols as prescribed by CHARMM-GUI, and then replaced the CHARMM36 additive force field [1] with the Stockholm lipids (Slipids) force field [4] for lipids. We performed at least a 100 ns long NpT simulation of each membrane with the Slipids force field.

There are two available crystal structures for bovine NPC2: *apo* (PDB ID: 1NEP [8]) and CHOL-

bound (PDB ID: 2HKA [9], chains B and C). Intrinsic pK_a of each residue was estimated for the crystal structure of the *apo* form and all three chains found in the crystal structure of using PROPKA 3.1 [10, 11]. Accordingly, E110, H31, and H56, which have intrinsic pK_a > 5.0, were protonated. One of the cholesterol-bound chains (chain C of PDB ID: 2HKA [8]) and the *apo* form (PDB ID: 1NEP [9]) were placed in 0.1 M NaCl solution. After successive minimization and 100 ps long NVT and NpT simulations with the protein and ligand heavy atoms harmonically restrained ($k = 100\,\mathrm{kJ/mol/Å^2}$), we performed 100 ns long NpT simulations for NPC2^{apo} and NPC2$^{\text{CHOL-bound}}$.

The simulation systems presented in this work were prepared by stacking the final configurations from the equilibrated membrane and protein systems. After combining the systems, excess water was removed and the ionic concentration was re-adjusted to 0.1 M. Random initial orientations of the protein with respect to the membrane were obtained by rotating a sphere that contains the protein and a layer of solvent around a random axis by 180°. These systems were then minimized and a 100 ps NpT simulation was performed with the protein heavy atoms harmonically restrained ($k = 100\,\mathrm{kJ/mol/Å^2}$). We then performed 400 ns long unbiased simulations for the systems indicated in Table 1.

S1.3 Well-tempered metadynamics simulations

S1.3.1 Simulation approach

All free energy calculations presented in this work use WT-MTD [12] with three collective variables (CVs). These three CVs capture different positions and orientations of the protein with respect to the membrane surface. The first CV, $|z|$, is the absolute value of the z-component of the distance between the protein center of mass (COM) and the COM of P atoms of the upper leaflet of the bilayer. The other CVs, θ and ϕ, are defined as the angles between the membrane normal and the long and the short axes of the protein, respectively (Main text, Fig 2 A–B).

To limit the phase space, we biased each CV separately within overlapping windows (index w) flanked by half-harmonic flat-bottomed restraints on the $|z|$ in the form of

$$V_w^b = \begin{cases} k_w^{min}(|z| - |z|_w^{min})^2 & \text{, if } |z| < |z|_w^{min} \\ 0 & \text{, if } |z|_w^{min} \le |z| \le |z|_w^{max} \\ k_w^{max}(|z| - |z|_w^{max})^2 & \text{, if } |z| > |z|_w^{max}. \end{cases} \tag{1}$$

Main text, Fig 2 C shows a schematic representation of the sampling approach. Each window has a width ($|z|_w^{max} - |z|_w^{min}$) of 4 Å and overlap ($|z|_w^{max} - |z|_{w+1}^{min}$) of 1 Å with the neighboring window, and all force constants (k_w^{min}, k_w^{max}) were set to 150 kJ/mol/Å^2. Within each window w, we performed separate WT-MTD [12] on $|z|$, θ, and ϕ CVs. Therefore, in each simulation (index i), a history-dependent bias on one of $|z|$, θ, and ϕ CVs, as well as a time-independent flat-bottomed half-harmonic potential on $|z|$ (V_w^b) were active. Metadynamics bias on $|z|$ was applied only within the interval, $|z|_w^{min} \le |z| \le |z|_w^{max}$ [13], whereas the metadynamics (MTD) biases on θ and ϕ were active throughout the CV space. The width of Gaussian hills was set to 0.05 Å for $|z|$ and 0.05 rad for θ and ϕ CVs. The frequency of hill addition was 1 ps^{-1} for all simulations. Bias factor (γ) was adjusted early in the simulations and finally kept at 40 in every simulation. The history dependent

bias was stored on a grid with a spacing of 0.01 for computational efficiency [14]. The neighboring simulations i and $i+1$ were coupled through bias exchange scheme [13,15] to improve sampling and phase space overlap. Bias exchanges were attempted between neighbors in alternating even-odd patterns every 1 ps as indicated by arrows in main text, Fig 2 C.

We performed 51–54 (17–18 windows) 200 ns long simulations spanning a $|z|$ range from the surface of the membrane to bulk solvent for each system. Each simulation was initialized using a random configuration from unbiased simulations that conform to the $|z|$ limits of the window. In cases where no unbiased simulation was previously performed or a particular window could not be initialized from the unbiased simulation due to lack of sampling, we used short adiabatic bias molecular dynamics (ABMD) [16] simulations with a ratchet-and-pawl like restraint along $|z|$ (force constant of 150 kJ/mol/Å^2) to generate initial configurations starting from a nearby configuration.

Initial 10 ns of each trajectory was discarded and configurations saved at every 100 ps were reweighted combining a time-independent free energy estimator for MTD [17] and a modified version of weighted histogram analysis method (WHAM) [18] as described in Section S1.3.2. All free energy surfaces were reconstructed with sample weights ($p_{i,t}$) used in *adaptive two-stage weighted kernel density estimation* [19], where the bandwidths for the pilot estimate were determined according to Silverman's rule of thumb [20]. The statistical errors were estimated with the Bayesian block bootstrap approach [21,22] as described in Section S1.3.4, using each independent simulation as a single block from 100 bootstrap samples.

S1.3.2 Unbiasing the WT-MTD simulations

Here, we present a new protocol to unbias free energy simulations employing a combination of time-independent and MTD biases. Similar combinations of umbrella sampling (US) and MTD and protocols to obtain free energy surfaces was put forward elsewhere [23,24]. Our approach allows associating weights, $p_{i,t}$, with each sampled configuration $\mathbf{R}_{i,t}$ in simulation i at time t, simplifying the construction of PMFs as a function of arbitrary CVs and the estimation of equilibrium averages. To this end, we use the time-independent free energy estimator for MTD recently introduced by Tiwary and Parrinello [17] to reweight sampled configurations, lifting the time-dependence due to MTD. We then use these weights in the non-parametric variant of the Weighted Histogram Analysis Method (WHAM) introduced by Bartels and Karplus [25] to combine all simulations. This approach can be easily generalized to any acting combination of MTD-type and time-independent biases for other applications.

We first estimate the MTD weights associated with each configuration $\mathbf{R}_{i,t}$ based on ref. [17]:

$$p^m_{i,t} = \mathrm{e}^{\beta(V^m_{i,t} - c^m_{i,t})}, \tag{2}$$

where $V^m_{i,t}$ and $c^m_{i,t}$ are the instantaneous MTD bias and the bias offset, respectively. Recently, Tiwary and Parrinello introduced a time-independent locally-converging free energy estimator for the MTD, allowing calculation of $c^m(t)$ [17]. Changing their notation [17], the free energy estimate for a MTD simulation on the collective variables $\mathbf{s}$ can be expressed as

$$F(\mathbf{s}) = F'(\mathbf{s},t) + \beta^{-1}\ln\int \mathrm{d}\,\mathbf{s}\ \mathrm{e}^{-\beta F'(\mathbf{s},t)}, \tag{3}$$

where the instantaneous free energy estimate $F'(\mathbf{s},t) = \frac{\gamma}{1-\gamma} V^m(\mathbf{s},t)$ and γ is the MTD bias factor. Plugging Eq 3 into the expression for $c^m(t)$ [17] and simplifying, one can directly estimate the bias offset from the stored MTD kernels:

$$c^m(t) = \beta^{-1} \ln \frac{\int \mathrm{d}\,\mathbf{s}\ \mathrm{e}^{-\beta F(\mathbf{s})}}{\int \mathrm{d}\,\mathbf{s}\ \mathrm{e}^{-\beta(F(\mathbf{s})+V^m(\mathbf{s},t))}} = \beta^{-1} \ln \frac{\int \mathrm{d}\,\mathbf{s}\ \mathrm{e}^{-\beta F'(\mathbf{s},t)}}{\int \mathrm{d}\,\mathbf{s}\ \mathrm{e}^{-\frac{\beta}{\gamma} F'(\mathbf{s},t)}}. \tag{4}$$

Next, we reweight each configuration $\mathbf{R}_{i,t}$ with the associated weight $p^m_{i,t}$ before using the non-parametric WHAM equations introduced by Bartels and Karplus [25]. This can be achieved by modifying the equations in ref. [25] as follows and solving iteratively until convergence:

$$\begin{aligned} p_{i,t} &= p^m_{i,t} \times (\sum_j N'_j c^b_j\, \mathrm{e}^{-\beta V^b_j(\mathbf{R}_{i,t})})^{-1} \\ c^b_j &= (\sum_i \sum_{t=1}^{N_i} p_{i,t}\, \mathrm{e}^{-\beta V^b_j(\mathbf{R}_{i,t})})^{-1}, \end{aligned} \tag{5}$$

where $N'_j = \sum_{t=1}^{N_j} p^m_{j,t}$, and V^b_j is the time-independent bias function in simulation j evaluated for configuration $\mathbf{R}_{i,t}$.

The PMF can then be estimated as a function of arbitrary CVs $\mathbf{q}$ using:

$$\Delta G(\mathbf{q}) = -\beta^{-1} \ln \langle \delta(\mathbf{q'}(\mathbf{R}_{i,t}) - \mathbf{q}) \times p_{i,t} \rangle\,, \tag{6}$$

where the average is taken over all sampled configurations. The PMFs were reconstructed for various CVs using weighted kernel density estimation and normalized with respect to the aqueous phase. Similarly, reported free energy values associated with each orientation in Table 1 are obtained by

$$\Delta G_{ori.} = -\beta^{-1} \ln \frac{\sum_{R_{i,t} \in ori.} p_{i,t}/\Omega_{ori.}}{\sum_{R_{i,t} \in \mathrm{aq}} p_{i,t}/\Omega_{\mathrm{aq}}}, \tag{7}$$

where the sum in the numerator is taken over a subset of all sampled configurations forming a particular orientation ($ori.$:= [*Prone Supine*]) and the sum in the denominator is taken over configurations in the aqueous (aq) phase. *Prone* and *Supine* modes were defined based on the z–ϕ space as marked on S1 Fig B. The $\Omega_{ori.}$ and Ω_{aq} are normalization factors equal to the volume of the collective variable space used in the definition of the binding orientation, *ori.*, and the aqueous phase, respectively. Table 1 also shows the free energy contribution by membrane associated states defined as $5\,\text{Å} < z < 25\,\text{Å}$ collectively.

S1.3.3 Normalized contact frequency analysis

In this application, we define the normalized contact frequency (NCF) as follows:

$$NCF^{\mathrm{BMP/memb.}}_{r,ori.} = CF^{\mathrm{BMP}}_{r,ori.} / CF^{\mathrm{memb.}}_{r,ori.}, \tag{8}$$

where $CF^{\mathrm{BMP}}_{r,ori.}$ and $CF^{\mathrm{memb.}}_{r,ori.}$ denote the contact frequency of residue r with BMP and any membrane lipid in a particular orientation, respectively. The NCF are calculated for *Prone mode* and *Supine mode*, separately, to get $NCF^{\mathrm{BMP/memb.}}_{\mathrm{r},Prone\ mode}$ and $NCF^{\mathrm{BMP/memb.}}_{\mathrm{r},Supine\ mode}$. We assign configurations to *Prone*

mode and *Supine mode* based on the $\min z_r$–ϕ–θ space as marked on main text, Fig 3 B and $80^\circ < \theta < 100^\circ$. We averaged the NCFs over all BMP-membrane free energy simulations and calculate the standard deviation as shown in main text, Fig 4 A.

The contact frequency of residue r with any other group of atoms, g, can be calculated from the biased simulations as a weighted average

$$CF^{g}_{r,ori.} = \frac{\sum_{R_{i,t} \in ori.} C^{g}_{r,i,t} \times p_{i,t}}{\sum_{R_{i,t} \in ori.} p_{i,t}}, \tag{9}$$

where $C^{g}_{r,i,t}$ is a number associated residue, r, and configuration $R_{i,t}$ and the sums are taken over all configurations assigned to a particular orientation. In this application, r is a protein residue, g is either BMP or any lipid. $C^{r_1,r_2}_{i,t}$ is a binary number equal to one if any atom of g is within 3 Å of any atom of r in configuration $R_{i,t}$, and is equal to zero, otherwise.

S1.3.4 Error estimation using Bayesian block bootstrapping

The Bayesian block bootstrap protocol for error estimation can easily be implemented into Eq 5 by making the following assignment

$$p^{m}_{i,t} \leftarrow p^{m}_{i,t} \times w^{b}_{i,t}, \tag{10}$$

where $w^{b}_{i,t}$ represents a set of random weights drawn according to the Bayesian bootstrap protocol for bootstrap sample b [26]. In the block variant, we first group the configurations $\mathbf{R}_{i,t}$ into N blocks (each containing n_{block} samples) that are presumably independent and identically distributed. This can be done based on taking blocks of length equal to at least the autocorrelation time of the quantity of interest, or more conservatively taking each simulation as a single block. Then, N weights w^{b}_{block} are drawn randomly according to Bayesian bootstrap protocol and each configuration $\mathbf{R}_{i,t}$ belonging to the block is assigned the weight $w^{b}_{i,t} = w^{b}_{block}/n_{block}$. Finally, Eq 5 is solved with the modified $p^{m}_{i,t}$ to estimate $p^{b}_{i,t}$ associated with bootstrap sample b. With sufficiently many sets of p^{b}, equilibrium averages and PMFs can be calculated for each set and their standard deviation gives the error estimate.

References

1. Klauda JB, Venable RM, Freites JA, O'Connor JW, Tobias DJ, Mondragon-Ramirez C, et al. Update of the CHARMM All-Atom Additive Force Field for Lipids: Validation on Six Lipid Types. J Phys Chem B. 2010;114(23):7830–7843. doi:10.1021/jp101759q.

2. Jo S, Kim T, Iyer VG, Im W. CHARMM-GUI: A Web-Based Graphical User Interface for CHARMM. J Comput Chem. 2008;29(11):1859–1865. doi:10.1002/jcc.20945.

3. Abraham MJ, Murtola T, Schulz R, Páll S, Smith JC, Hess B, et al. GROMACS: High Performance Molecular Simulations through Multi-Level Parallelism from Laptops to Supercomputers. SoftwareX. 2015;1-2:19–25. doi:10.1016/j.softx.2015.06.001.

4. Jämbeck JPM, Lyubartsev AP. Another Piece of the Membrane Puzzle: Extending Slipids Further. J Chem Theory Comput. 2013;9(1):774–784. doi:10.1021/ct300777p.

5. Dupradeau FY, Pigache A, Zaffran T, Savineau C, Lelong R, Grivel N, et al. The R.E.D. Tools: Advances in RESP and ESP Charge Derivation and Force Field Library Building. Phys Chem Chem Phys. 2010;12(28):7821–7839. doi:10.1039/C0CP00111B.

6. Frisch MJ, Trucks GW, Schlegel HB, Scuseria GE, Robb MA, Cheeseman JR, et al.. Gaussian 09 Revision D.01;.

7. McCauliff LA. Cholesterol Transfer by NPC2 Protein and Cyclodextrins in Niemann Pick Type C Disease. Rutgers University - Graduate School - New Brunswick; 2014.

8. Friedland N, Liou HL, Lobel P, Stock AM. Structure of a Cholesterol-Binding Protein Deficient in Niemann-Pick Type C2 Disease. Proc Natl Acad Sci USA. 2003;100(5):2512–2517. doi:10.1073/pnas.0437840100.

9. Xu S, Benoff B, Liou HL, Lobel P, Stock AM. Structural Basis of Sterol Binding by NPC2, a Lysosomal Protein Deficient in Niemann-Pick Type C2 Disease. J Biol Chem. 2007;282(32):23525–23531. doi:10.1074/jbc.M703848200.

10. Søndergaard CR, Olsson MHM, Rostkowski M, Jensen JH. Improved Treatment of Ligands and Coupling Effects in Empirical Calculation and Rationalization of pK_a Values. J Chem Theory Comput. 2011;7(7):2284–2295. doi:10.1021/ct200133y.

11. Olsson MHM, Søndergaard CR, Rostkowski M, Jensen JH. PROPKA3: Consistent Treatment of Internal and Surface Residues in Empirical pK_a Predictions. J Chem Theory Comput. 2011;7(2):525–537. doi:10.1021/ct100578z.

12. Barducci A, Bussi G, Parrinello M. Well-Tempered Metadynamics: A Smoothly Converging and Tunable Free-Energy Method. Phys Rev Lett. 2008;100(2). doi:10.1103/PhysRevLett.100.020603.

13. Baftizadeh F, Cossio P, Pietrucci F, Laio A. Protein Folding and Ligand-Enzyme Binding from Bias-Exchange Metadynamics Simulations. Curr Phys Chem. 2012;2(1):79–91. doi:10.2174/1877946811202010079.

14. Babin V, Roland C, Sagui C. Adaptively Biased Molecular Dynamics for Free Energy Calculations. J Chem Phys. 2008;128(13):134101. doi:10.1063/1.2844595.

15. Marinelli F, Pietrucci F, Laio A, Piana S. A Kinetic Model of Trp-Cage Folding from Multiple Biased Molecular Dynamics Simulations. PLoS Comput Biol. 2009;5(8):e1000452. doi:10.1371/journal.pcbi.1000452.

16. Marchi M, Ballone P. Adiabatic Bias Molecular Dynamics: A Method to Navigate the Conformational Space of Complex Molecular Systems. J Chem Phys. 1999;110(8):3697–3702. doi:10.1063/1.478259.

17. Tiwary P, Parrinello M. A Time-Independent Free Energy Estimator for Metadynamics. J Phys Chem B. 2015;119(3):736–742. doi:10.1021/jp504920s.

18. Moradi M, Enkavi G, Tajkhorshid E. Atomic-Level Characterization of Transport Cycle Thermodynamics in the Glycerol-3-Phosphate:Phosphate Antiporter. Nat Commun. 2015;6:8393. doi:10.1038/ncomms9393.

19. Van Kerm P. Adaptive Kernel Density Estimation. Stata J. 2003;3(2):148–156.

20. Silverman BW. Density Estimation for Statistics and Data Analysis. vol. 26. CRC press; 1986.

21. Hub JS, de Groot BL, van der Spoel D. g_wham—a Free Weighted Histogram Analysis Implementation Including Robust Error and Autocorrelation Estimates. J Chem Theory Comput. 2010;6(12):3713–3720. doi:10.1021/ct100494z.

22. Moradi M, Tajkhorshid E. Computational Recipe for Efficient Description of Large-Scale Conformational Changes in Biomolecular Systems. J Chem Theory Comput. 2014;10(7):2866–2880. doi:10.1021/ct5002285.

23. Johnston JM, Wang H, Provasi D, Filizola M. Assessing the Relative Stability of Dimer Interfaces in G Protein-Coupled Receptors. PLoS Comput Biol. 2012;8(8):e1002649. doi:10.1371/journal.pcbi.1002649.

24. Awasthi S, Kapil V, Nair NN. Sampling Free Energy Surfaces as Slices by Combining Umbrella Sampling and Metadynamics. J Comput Chem. 2016;37(16):1413–1424. doi:10.1002/jcc.24349.

25. Bartels C. Analyzing Biased Monte Carlo and Molecular Dynamics Simulations. Chem Phys Lett. 2000;331(5-6):446–454. doi:10.1016/S0009-2614(00)01215-X.

26. Rubin DB. The Bayesian Bootstrap. Ann Stat. 1981;9(1):130–134. doi:10.1214/aos/1176345338.
