# Supplementary figures and images for: Concerted regulation of npc2 binding to endosomal/lysosomal membranes by bis(monoacylglycero)phosphate and sphingomyelin
Source: PLoS Comput Biol. 2017 Oct 30;13(10):e1005831. doi: 10.1371/journal.pcbi.1005831 (PMC5679659; doi:10.1371/journal.pcbi.1005831)

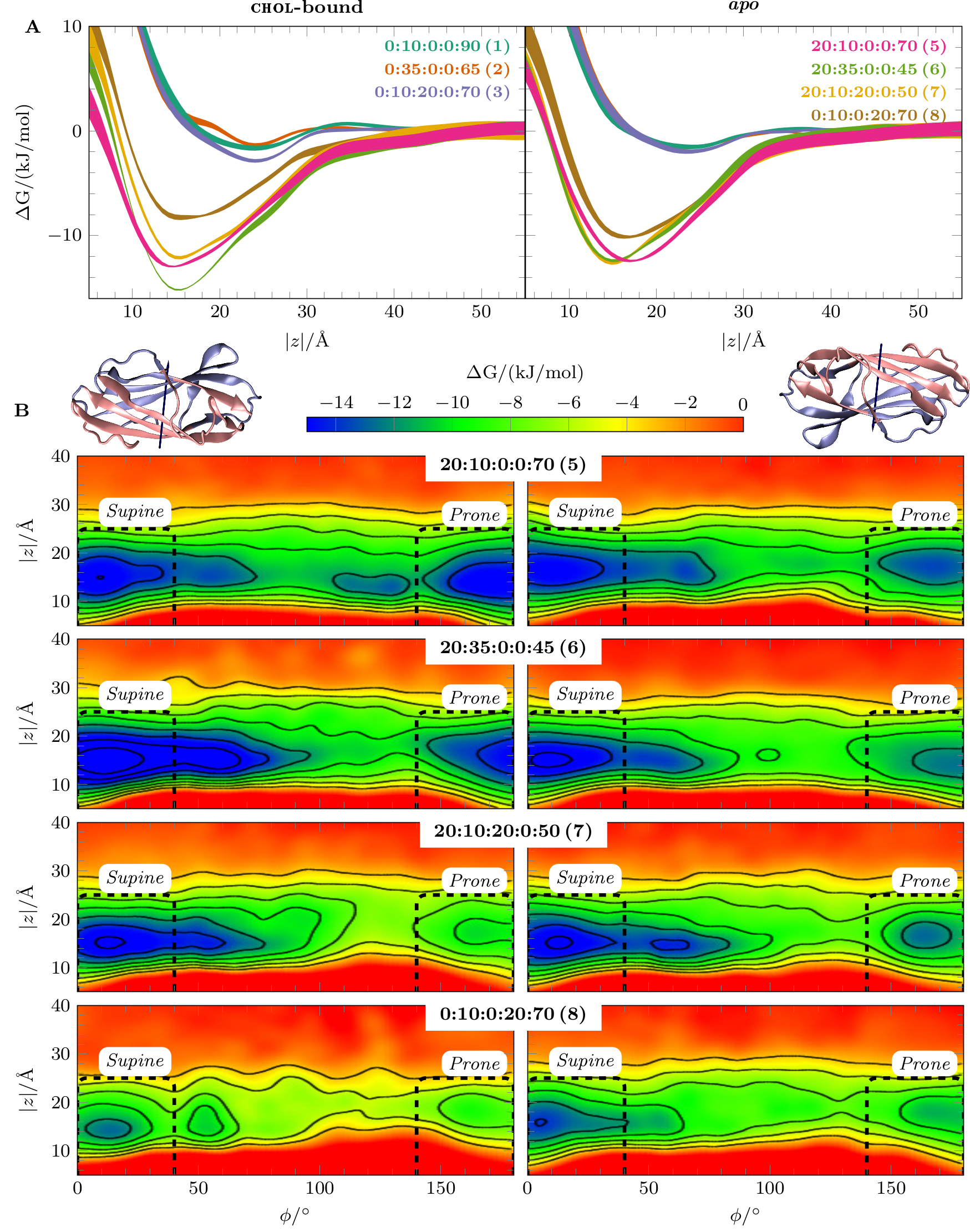

Supplement: S1 Fig — The free energy surfaces for cholesterol-bound (left column) and apo (right column) npc2 binding to membranes with different compositions. A) Potential of mean force (pmf) profiles shown as a function of |z|. The band thickness displays the error. B) pmf surfaces shown as a function of |z| and ϕ. Contours were plotted every 2 kJ/mol increments. The two binding orientations are marked with dashed lines. The thumbnail images of npc2 structure for the corresponding orientations are shown on the upper left for Prone mode and upper right for Supine mode. The labels indicate membrane content in molar fractions for a mixture of bmp:chol:sm:dopg:popc, and the corresponding system numbers (Table 1) are provided in parentheses. The local errors are given in S2 Fig. (TIFF) [file pcbi.1005831.s002.tiff]

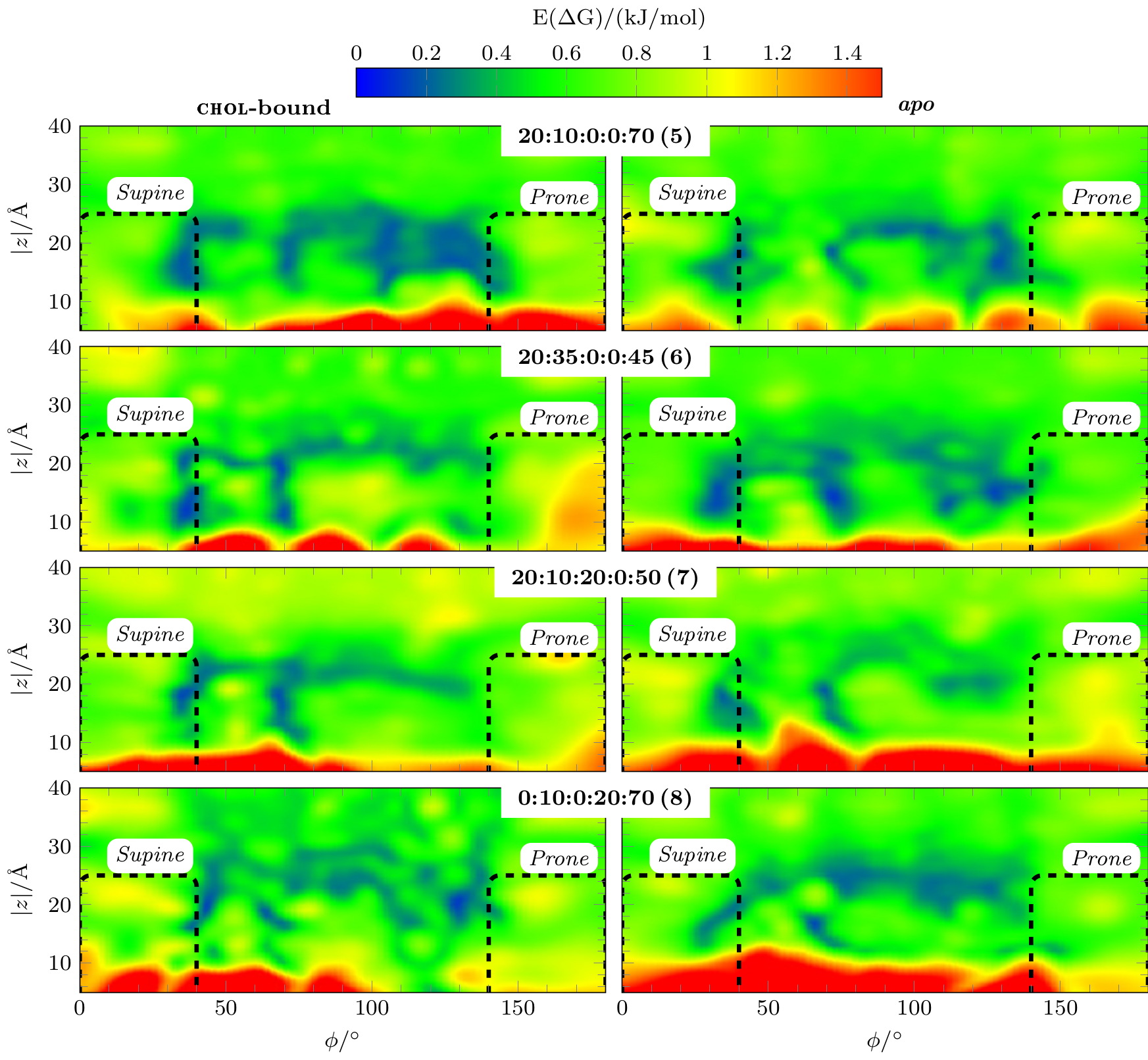

Supplement: S2 Fig — Local errors of |z| vs. ϕ free energy surfaces for cholesterol-bound (left column) and apo (right column) npc2 binding to anionic membranes with indicated compositions. The two binding orientations are marked with dashed lines. The labels indicate membrane content in molar fractions for a mixture of bmp:chol:sm:dopg:popc, and the corresponding system numbers (Table 1) are provided in parentheses. (TIFF) [file pcbi.1005831.s003.tiff]

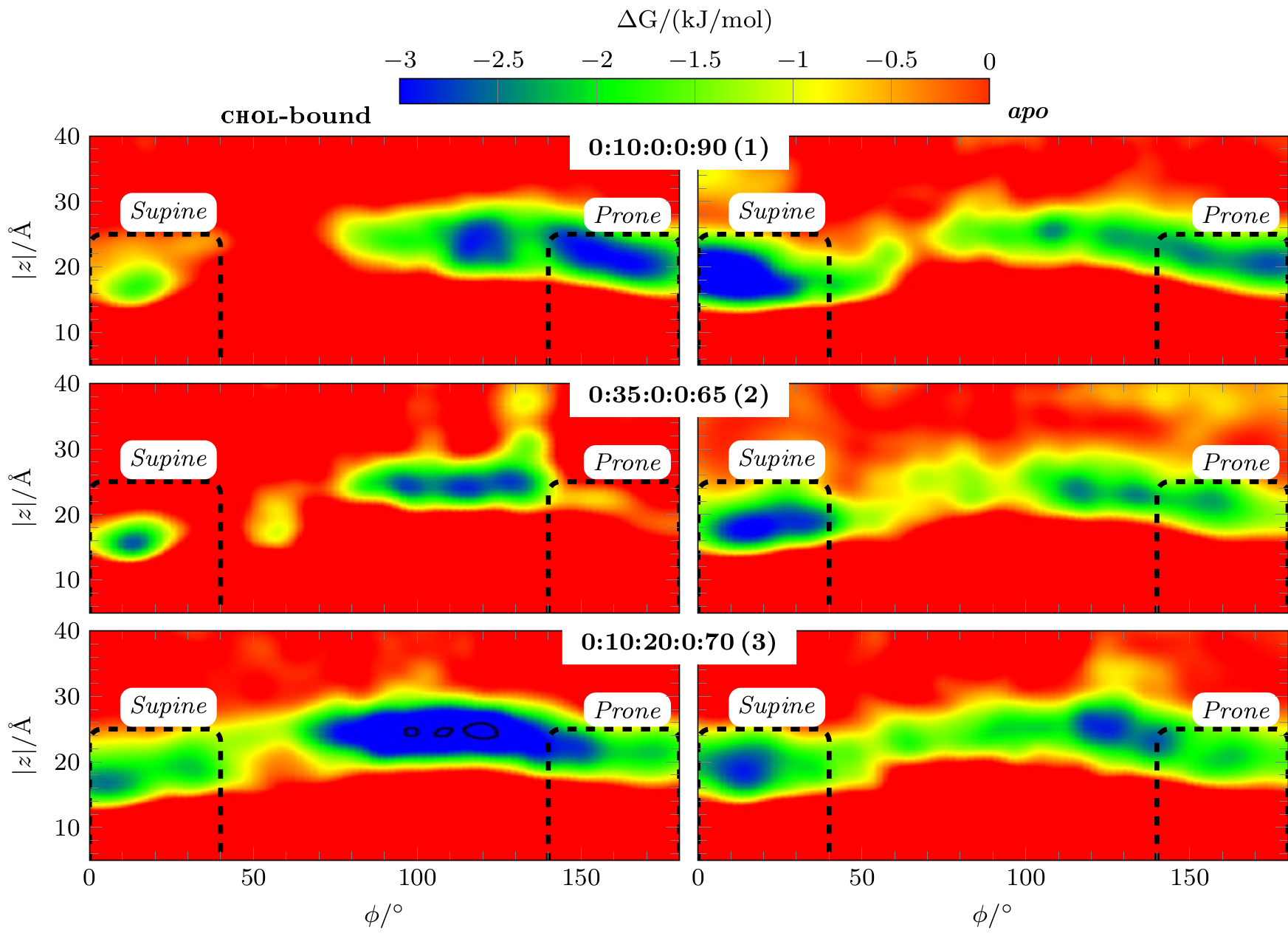

Supplement: S3 Fig — The |z| vs. ϕ free energy surfaces for cholesterol-bound (left column) and apo (right column) npc2 binding to neutral membranes with indicated compositions. The two binding orientations are marked with dashed lines. The labels indicate membrane content in molar fractions for a mixture of bmp:chol:sm:dopg:popc, and the corresponding system numbers (Table 1) are provided in parentheses. The local errors are given in S4 Fig. (TIFF) [file pcbi.1005831.s004.tiff]

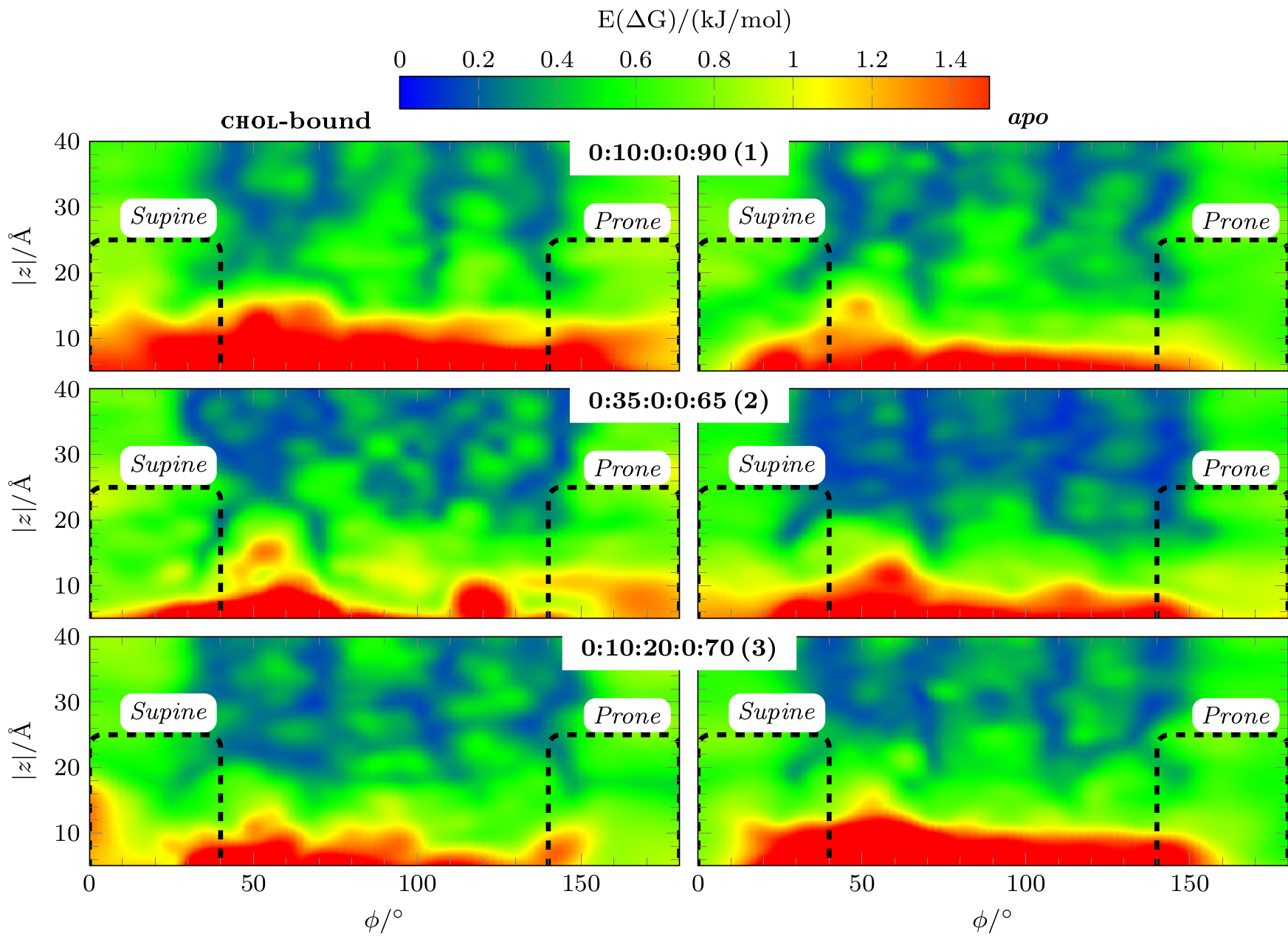

Supplement: S4 Fig — Local errors of |z| vs. ϕ free energy surfaces for cholesterol-bound (left column) and apo (right column) npc2 binding to neutral membranes with indicated compositions. The two binding orientations are marked with dashed lines. The labels indicate membrane content in molar fractions for a mixture of bmp:chol:sm:dopg:popc, and the corresponding system numbers (Table 1) are provided in parentheses. (TIFF) [file pcbi.1005831.s005.tiff]

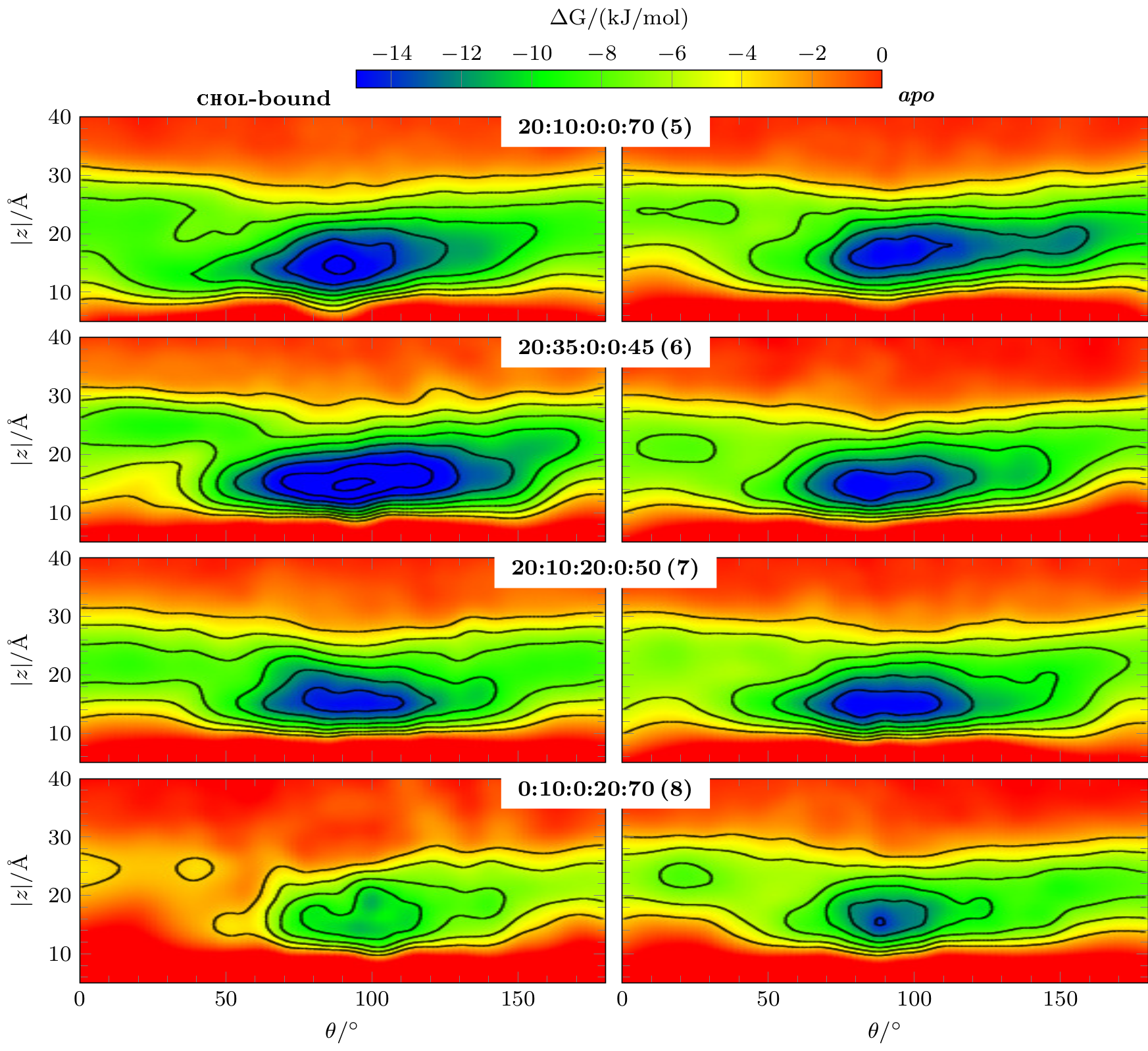

Supplement: S5 Fig — The |z| vs. θ free energy surfaces for cholesterol-bound (left column) and apo (right column) npc2 binding to anionic membranes with indicated compositions. The labels indicate membrane content in molar fractions for a mixture of bmp:chol:sm:dopg:popc, and the corresponding system numbers (Table 1) are provided in parentheses. The local errors are given in S6 Fig. (TIFF) [file pcbi.1005831.s006.tiff]

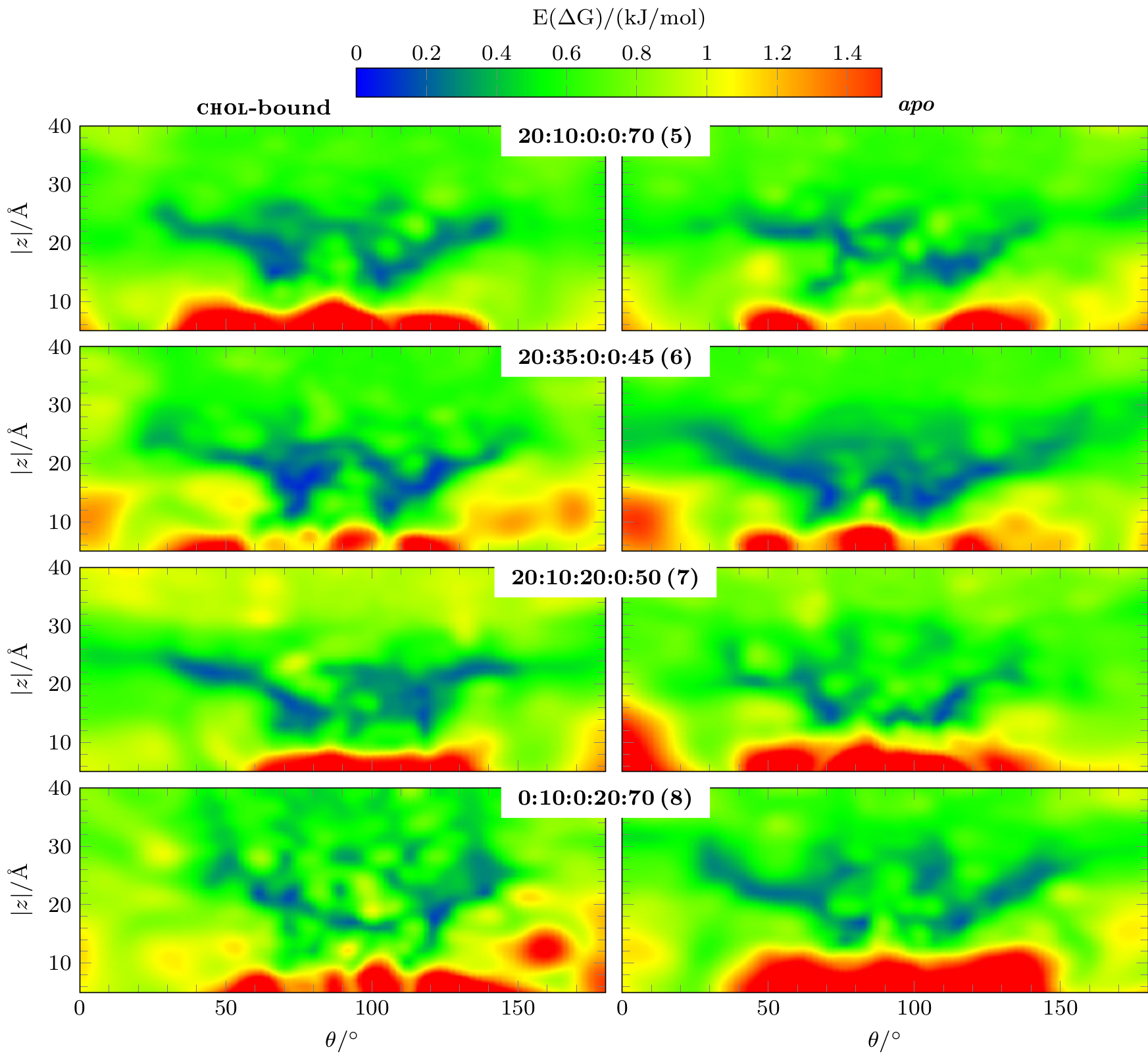

Supplement: S6 Fig — Local errors of |z| vs. θ free energy surfaces for cholesterol-bound (left column) and apo (right column) npc2 binding to anionic membranes with indicated compositions. The labels indicate membrane content in molar fractions for a mixture of bmp:chol:sm:dopg:popc, and the corresponding system numbers (Table 1) are provided in parentheses. (TIFF) [file pcbi.1005831.s007.tiff]

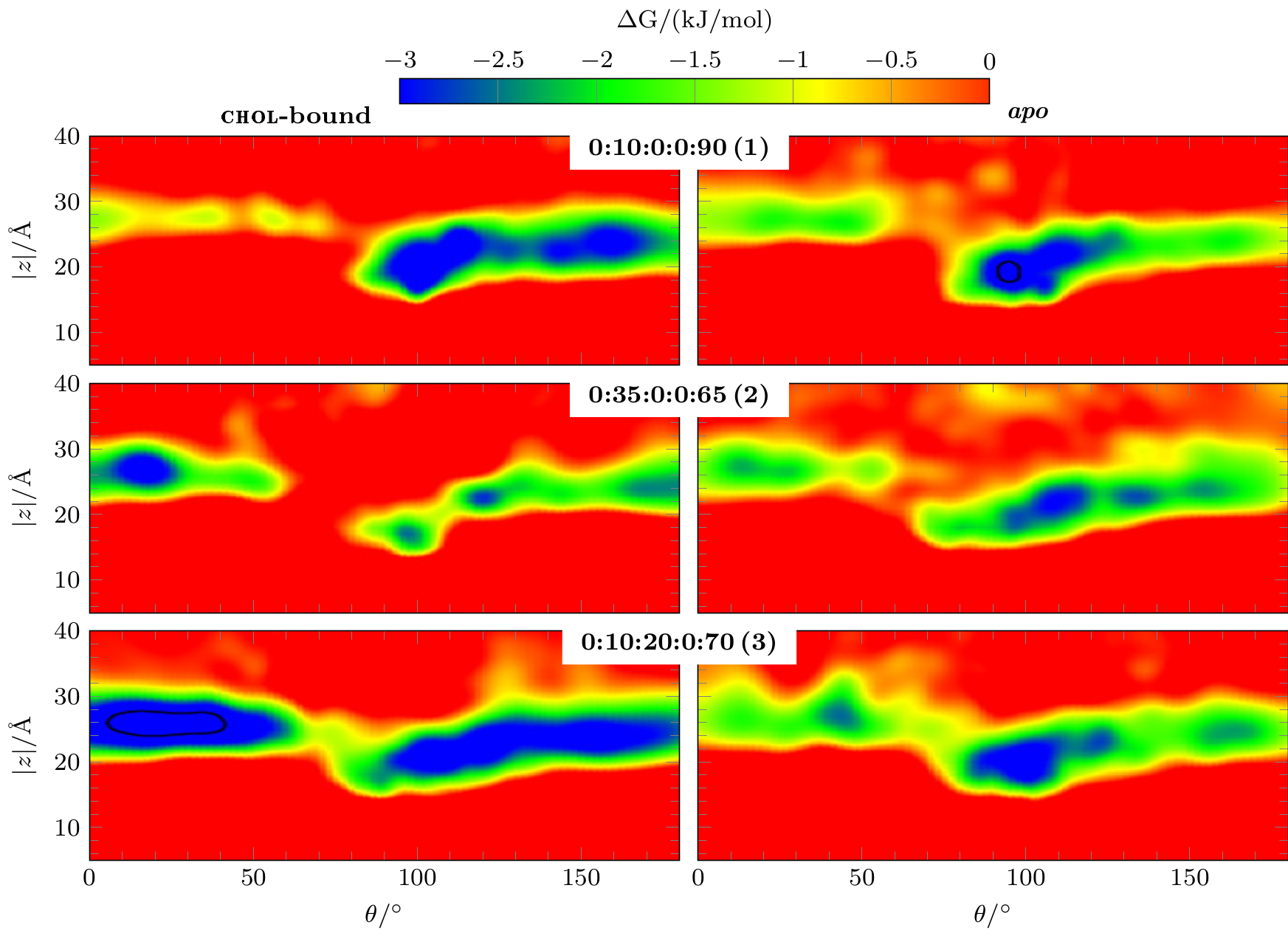

Supplement: S7 Fig — The |z| vs. θ free energy surfaces for cholesterol-bound (left column) and apo (right column) npc2 binding to neutral membranes with indicated compositions. The labels indicate membrane content in molar fractions for a mixture of bmp:chol:sm:dopg:popc, and the corresponding system numbers (Table 1) are provided in parentheses. The local errors are given in S8 Fig. (TIFF) [file pcbi.1005831.s008.tiff]

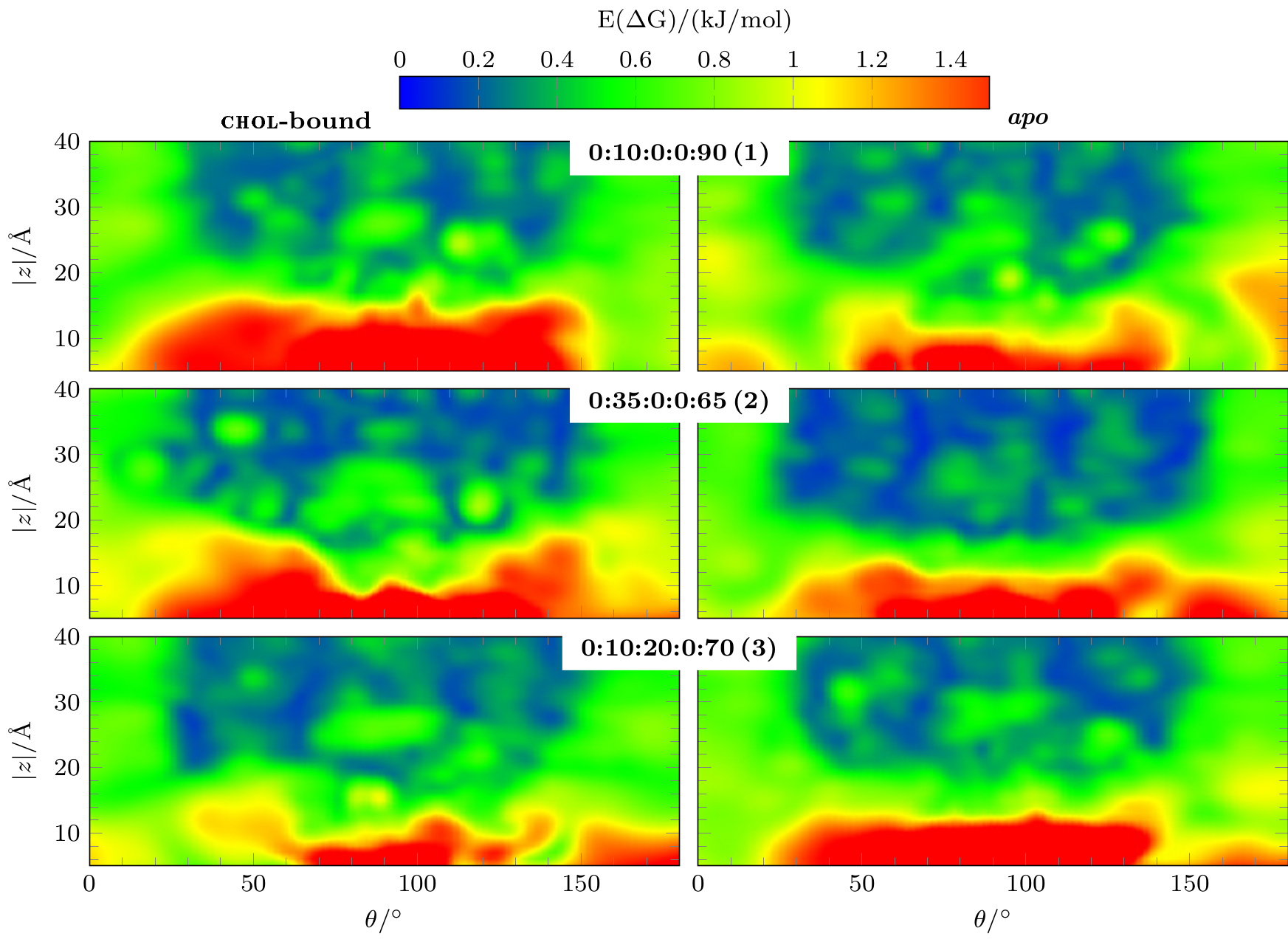

Supplement: S8 Fig — Local errors of |z| vs. θ free energy surfaces for cholesterol-bound (left column) and apo (right column) npc2 binding to neutral membranes with indicated compositions. The labels indicate membrane content in molar fractions for a mixture of bmp:chol:sm:dopg:popc, and the corresponding system numbers (Table 1) are provided in parentheses. (TIFF) [file pcbi.1005831.s009.tiff]

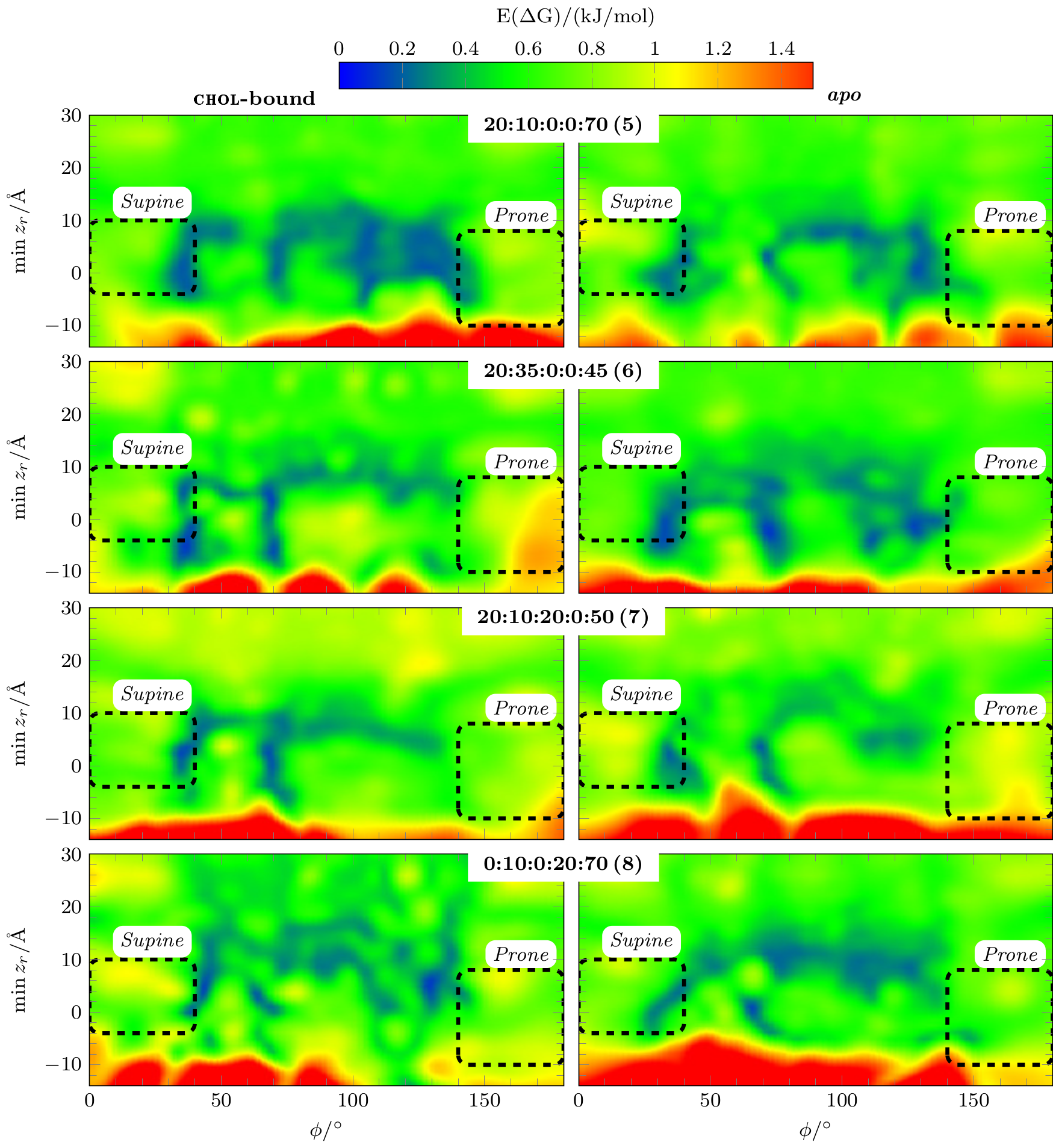

Supplement: S9 Fig — Local errors of min zr vs. ϕ free energy surfaces for cholesterol-bound (left column) and apo (right column) npc2 binding to charged membranes with indicated compositions. The two binding orientations are marked with dashed lines. The labels indicate membrane content in molar fractions for a mixture of bmp:chol:sm:dopg:popc, and the corresponding system numbers (Table 1) are provided in parentheses. (TIFF) [file pcbi.1005831.s010.tiff]

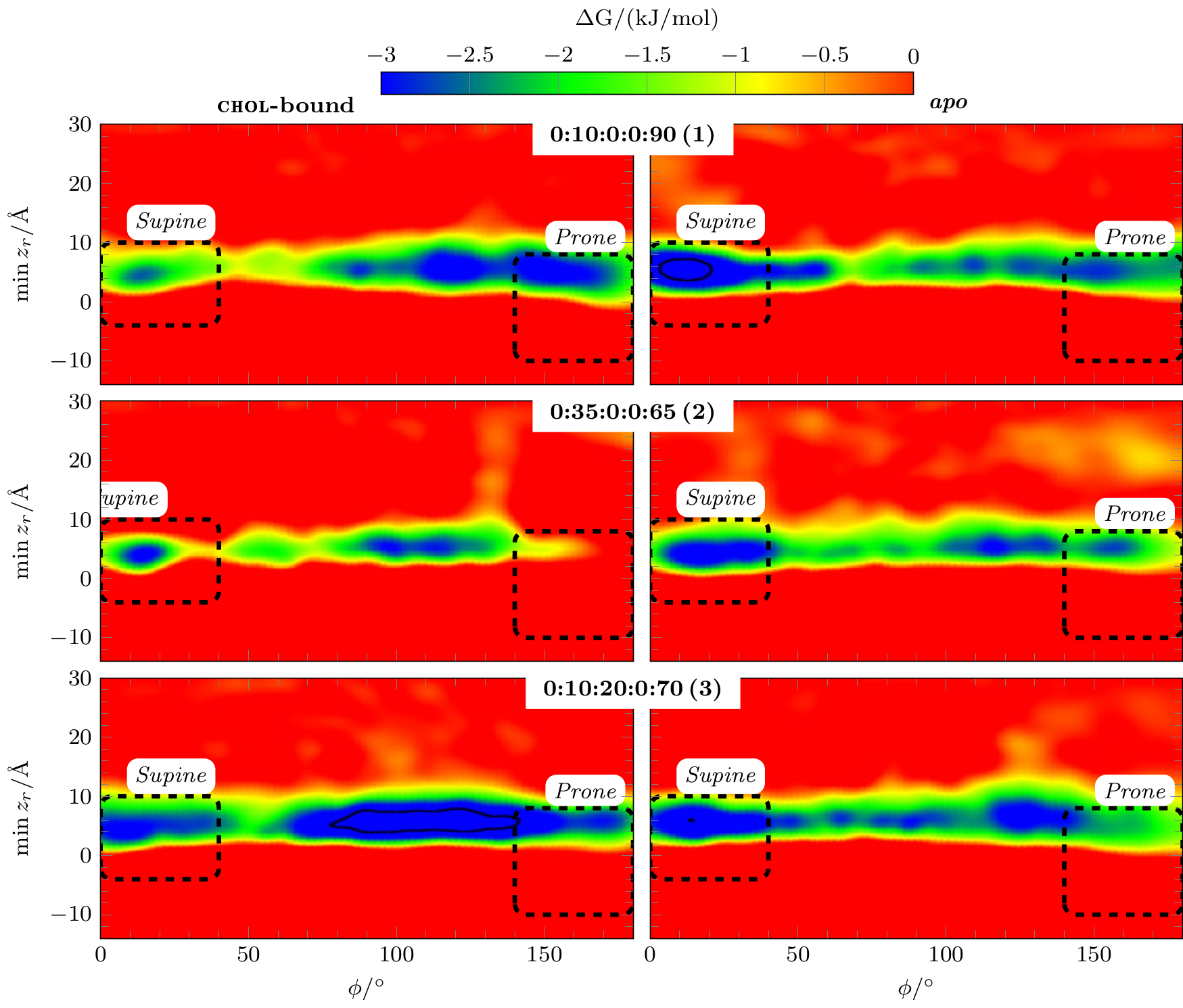

Supplement: S10 Fig — The min zr vs. ϕ free energy surfaces for cholesterol-bound (left column) and apo (right column) npc2 binding to neutral membranes with the indicated compositions. The two binding orientations are marked with dashed lines. The labels indicate membrane content in molar fractions for a mixture of bmp:chol:sm:dopg:popc, and the corresponding system numbers (Table 1) are provided in parentheses. The local errors are given in S11 Fig. (TIFF) [file pcbi.1005831.s011.tiff]

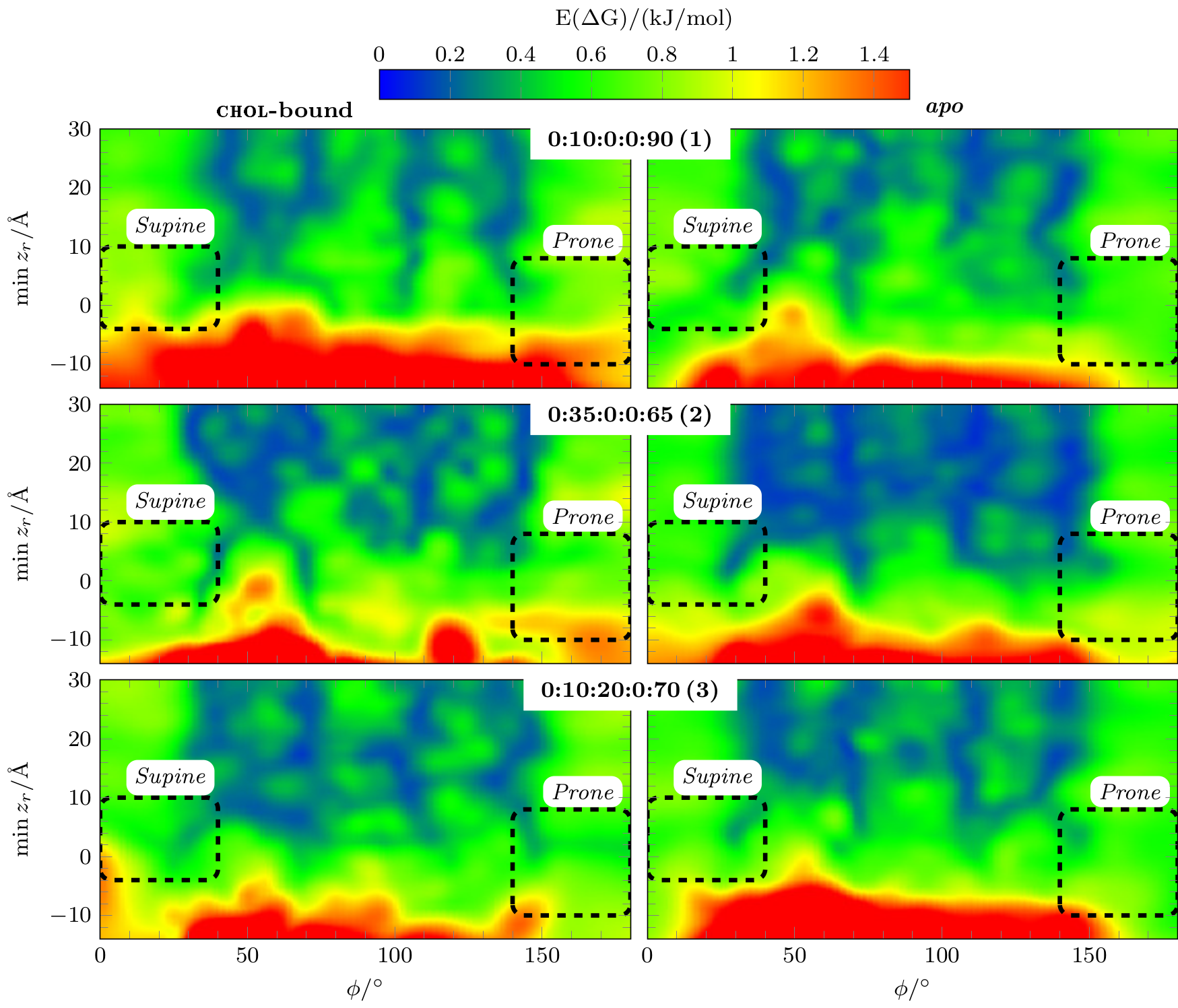

Supplement: S11 Fig — Local errors of min zr vs. ϕ free energy surfaces for cholesterol-bound (left column) and apo (right column) npc2 binding to neutral membranes with indicated compositions. The two binding orientations are marked with dashed lines. The labels indicate membrane content in molar fractions for a mixture of bmp:chol:sm:dopg:popc, and the corresponding system numbers (Table 1) are provided in parentheses. (TIFF) [file pcbi.1005831.s012.tiff]

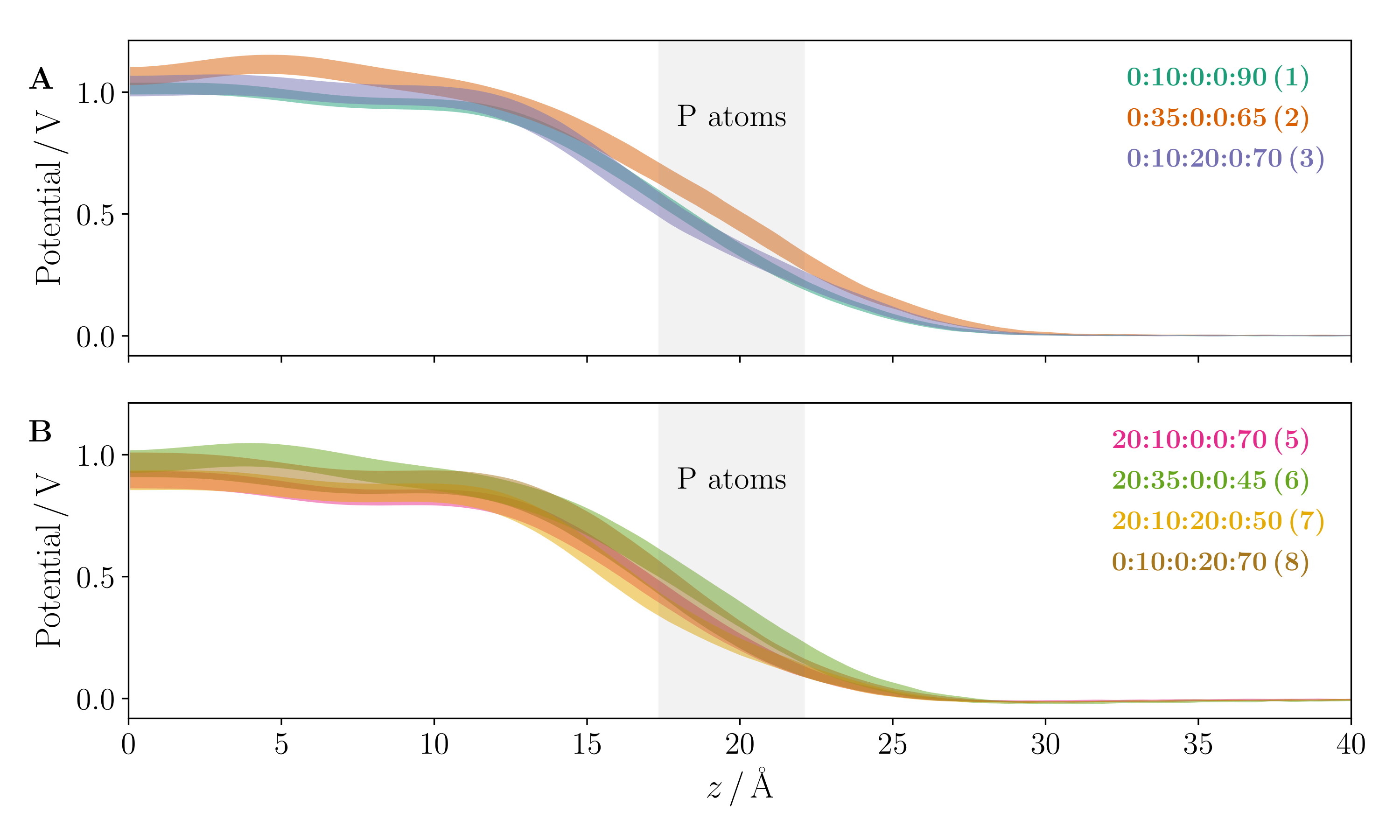

Supplement: S12 Fig — Electrostatic potential profiles for the neutral (A) and charged (B) membranes. The profiles are calculated from free energy simulation trajectories (the last 100 ns), where the protein (npc2apo) is kept at a non-interacting distance to the membrane (i = 45–53, Fig 2) to ensure that membranes are unaffected by the protein. The trajectories were first centered based membrane com and then, the electric field calculated by gmx potential tool included in gromacs 5.0 [29] is averaged over the upper and lower halves of the box for symmetry. The electric field is then integrated to get the electrostatic potential profiles for each simulation. The profiles were averaged over all relevant simulations of a particular system and the band thickness displays their standard error. The location of the membrane P atoms is indicated with gray bands. (TIFF) [file pcbi.1005831.s013.tiff]
